# Ensemble effort estimation with metaheuristic hyperparameters and weight optimization for achieving accuracy

**DOI:** 10.1371/journal.pone.0300296

**Published:** 2024-04-04

**Authors:** Anum Yasmin, Wasi Haider Butt, Ali Daud

**Affiliations:** 1 Department of Computer and Software Engineering, College of Electrical and Mechanical Engineering, National University of Sciences and Technology (NUST), Islamabad, Pakistan; 2 Faculty of Resilience, Rabdan Academy, Abu Dhabi, United Arab Emirates; National Institute of Technology Srinagar, INDIA

## Abstract

Software development effort estimation (SDEE) is recognized as vital activity for effective project management since under or over estimating can lead to unsuccessful utilization of project resources. Machine learning (ML) algorithms are largely contributing in SDEE domain, particularly ensemble effort estimation (EEE) works well in rectifying bias and subjectivity to solo ML learners. Performance of EEE significantly depends on hyperparameter composition as well as weight assignment mechanism of solo learners. However, in EEE domain, impact of optimization in terms of hyperparameter tunning as well as weight assignment is explored by few researchers. This study aims in improving SDEE performance by incorporating metaheuristic hyperparameter and weight optimization in EEE, which enables accuracy and diversity to the ensemble model. The study proposed *Metaheuristic-optimized Multi-dimensional bagging scheme and Weighted Ensemble (MoMdbWE)* approach. This is achieved by proposed search space division and hyperparameter optimization method named *as Multi-dimensional bagging (Mdb)*. Metaheuristic algorithm considered for this work is Firefly algorithm (FFA), to get best hyperparameters of three base ML algorithms (Random Forest, Support vector machine and Deep Neural network) since FFA has shown promising results of fitness in terms of MAE. Further enhancement in performance is achieved by incorporating FFA-based weight optimization to construct Metaheuristic-optimized weighted ensemble (MoWE) of individual multi-dimensional bagging schemes. Proposed scheme is implemented on eight frequently utilized effort estimation datasets and results are evaluated by 5 error metrices (MAE, RMSE, MMRE, MdMRE, Pred), standard accuracy and effect size along with Wilcox statistical test. Findings confirmed that the use of FFA optimization for hyperparameter (with search space sub-division) and for ensemble weights, has significantly enhanced performance in comparison with individual base algorithms as well as other homogeneous and heterogenous EEE techniques.

## 1. Introduction

Software development effort estimation (SDEE) is principle yet complex activity of software project management. Resource planning, budget allocation and other software management task can be affected adversely due to under or over software effort estimation [1]. Attaining accurate effort prediction is challenging, regardless of any software lifecycle model, particularly during the early phases of software project The reason is, there is lack of necessary details about future prospects of software projects and difficulty level it entails. This vagueness sometimes leads to unjustified and incorrect budget, human resources and schedule assignments, eventually leading to project failure. Accurate software estimate directs the project towards better planning, effective resource utilizations, and successful project deadlines. This necessitates the inclusion of automated effort estimation system which can contribute in fast decision-making for resources allocation [2, 3].

Several SDEE techniques have been proposed in past since early 1980s [4–9] which can be grouped into three broad categories [10]: (1) expert judgement [4]; requires past experience of similar projects in estimation; (2) parametric techniques [7] which are formula-based methods and generate function to estimate effort, based on a statistical analysis of historical projects; and (3) ML techniques [11] which utilize artificial intelligence algorithms to predict the development effort.

Traditionally, SDEE is accomplished using expert judgment, analogy, decomposition/recomposition, and parametric approaches, among which expert judgment is considered the most relied upon technique. However, the statistical and ML techniques can provide preliminary set of effort assessments to the experts which can support final verdict of expert estimation. Moreover, software effort and cost estimation via traditional approaches tend to be inaccurate since it is prone to human biasness and subjectivity [11, 12]. Hence, approaches based on human judgement could not provide correct estimate for diverse nature of development methods, technology and software industry. To deal with this problem, machine learning (ML) methods are appropriate to use, which are free from human biases and adaptable to normal project lifecycle changes. This fact is also supported by past literature of ML based SDEE studies, verifying the performance capabilities of intelligent methods. A systematic literature review (SLR) conducted by Jorgensen and Shepperd [7] analyzed 304 SDEE studies published before 2004. The authors identified that overall, 11 effort estimation techniques are explored in literature, among which 49% were regression models. Similar SLR work on ML SDEE is conducted by Wen at al. [11], which is based on 84 researches, published between 1990 and 2010. SLR concluded that ML techniques are drawn to give more accurate results as compared to non-ML or parametric techniques (i.e. expert judgment, function point analysis COCOMO and SLIM). Moreover, the study also revealed that, are decision trees (DT), support vector machine (SVR), case-based reasoning (CBR) and artificial neural networks (ANN) are frequently utilized ML models to estimate effort, and gained more attention over the periods of time [11].

Apart from the invent of multiple SDEE techniques, no particular method can be declared to perform better than the others under all circumstances. Researchers claimed that single SDEE techniques provide variable accuracy across different settings and failed to give correct estimate for multiple contexts. For eliminating this issue, using more than one technique and accumulating the results can significantly improve the estimation accuracy [1, 5, 13]. Ensemble effort estimation (EEE) techniques support this perceptive, which have been investigated recently in SDEE. EEE works by integrating more than one single techniques with a combination rule to predict effort of a new software project [14, 15]. An SLR conducted by Idri et al. [6] on EEE identified that ensemble estimation outperformed their respective solo learners in most of the studies. EEE techniques can be categorized into two groups [6, 10]: (1) Homogeneous EEE, which combines single SDEE techniques by (a) combining more than two configurations of the same technique, (b) by combining one single SDEE technique and one meta model [16] such as: Bagging [8], Boosting [17], Random Subspace and Negative Correlation [18] (2) Heterogeneous EEE in which at least two different SDEE single techniques are combined via some combination rule [6].

Moreover, obtaining high performance when using ensemble depends on two criteria [19, 20]: accuracy and diversity. Generating diversity refers to creating difference among individual learners, which is a fundamental concern in ensemble methods (Zhou, 2012). Diversity is regarded as significant attribute of a robust ensemble model, where ensemble constituents generate different errors for same input. Another perspective of diversity is when predictions made by each ensemble member are independent and uncorrelated [21]. Ensemble yields better results when created with diverse single techniques. Similarly, accuracy defines how well participants of ensemble predict effort for unseen instances. Accuracy of any ML algorithm is directly influenced by its hyperparameter settings. Hence, one way of achieving high accuracy is optimizing hyperparameters configuration of each single techniques according to the context on which algorithm is applied. Most of heterogenous EEE studies have relied on using same set of hyperparameters for solo learner on all datasets. This may introduce bias in results since, particular SDEE may generate different performance accuracy under one configuration and works entirely different under others [10]. Hence, hyperparameter optimization needs to be applied on ML model, adapting them to the current context. Hyperparameter optimization or automated hyperparameters’ tuning reduces the manual overhead of exploring various possible configuration settings of ML algorithm, in turn, improves the accuracy and reproducibility of SDEE model [19].

Metaheuristic optimization (MO) is recently investigated in ML based SDEE. Although, hyperparameter optimization for EEE is incorporated in previous SDEE studies, but the optimization impact on each base algorithm is only explored by few [3, 22, 23]. Further, in any of the SDEE hyperparameter optimization work, no clear discussion is made on search-space utilization to get best hyperparameters from. Search-space selection plays a vital part in optimization problem as it determines the area from which best parameters are extracted [24].

**Fig 1:**
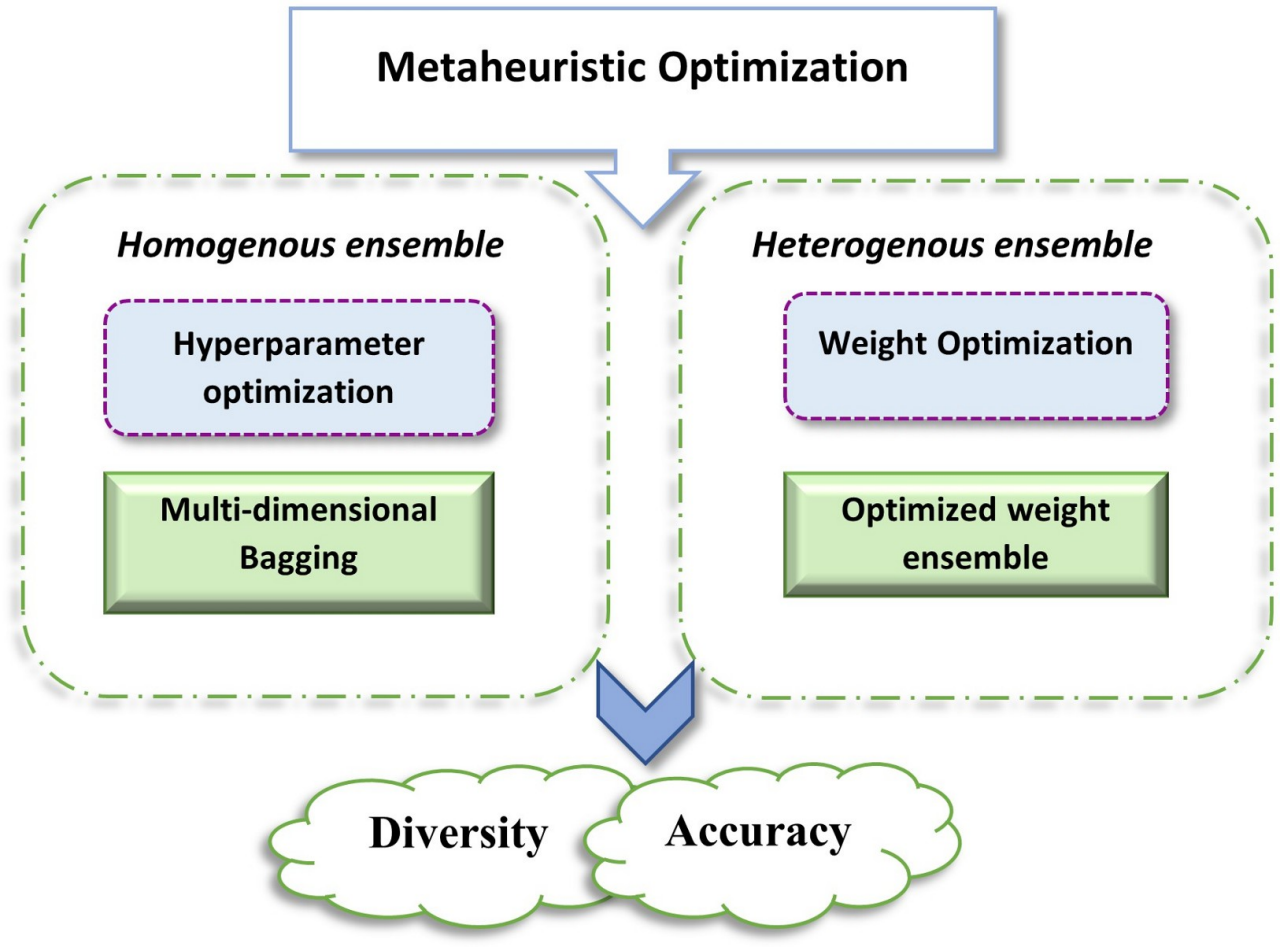
An illustration of proposed scheme.

This study aims to contribute in the field of SDEE by incorporating metaheuristic optimization in ensemble learning. Three base ML algorithms Random Forest (RF), Support Vector Machine (SVR), and Deep neural network (DeepNet) are employed for ensemble creation. The rationale behind choosing these ML techniques is that they are frequently incorporated solo learners in previous ensemble estimation work [6]. The study incorporates both ensemble types i.e., Homogeneous and Heterogenous ensemble. homogeneous ensemble is created with proposed *multi-dimensional bagging* with hyperparameter optimization. Search-space division is utilized for achieving best hyperparameters from each section of search-space. Multi-dimensional bagging schemes of three base algorithms are combined by Metaheuristic-optimized weights, favoring heterogenous ensemble. Fig 1 represents an illustrative overview of the work. Main contributions of this work are:

- Analyzing the impact of both homogeneous and heterogenous ensemble simultaneously in SDEE for better effort prediction.
- Performing hyperparameter search-space division for each base ML algorithm and getting optimal hyperparameters from each sub-space. This will induce accuracy and diversity to the model (basic requirement of ensemble implementation).
- Incorporating metaheuristics for: (a) hyperparameter optimization of each base ML algorithms, (b) assigning optimal weights to individual base algorithm for ensemble creation.

The rest of the paper is organized as follows; Section 2: Related work, presents previous SDEE work, in domain of ensemble effort estimation (Section 2.1: Ensemble effort estimation (EEE)) and metaheuristic optimization (Section 2.2: Metaheuristic optimization (MO)); also it describes the limitations identifies from the literature (Section 2.3: Summary of literature work limitations) and problem formulation presented by this study (Section 2.4: Problem formulation and research questions). Section 3: Background, describes theoretical and technical details of ML and metaheuristic techniques used in this study. Section 4: Methodology, explains proposed framework for creating Homogeneous and Heterogenous ensemble. Section 5: Experimental setup, contains details, including implementation of proposed framework (Section 5.1: Multi-dimensional bagging (Mdb): Implementation setup); dataset used in this study (Section 5.2: Datasets) and models compared with proposed scheme (Section 5.3: Comparison models). Performance evaluation (Section 5.4: Performance evaluation metrices) and statistical test (Section 5.5: Statistical evaluation) used in the study are elaborated next. Section 6: Results presents results achieved in this study and Section 7: Discussion contains discussion on problem formulations and proposed solution achieved in this work. Threats to validity and conclusion with future work are listed in Section 8: Threats to validity and Section 9: Conclusion and future work respectively.

## 2. Related work

Various SDEE studies are largely incorporating ML models, due to their inherent ability to derive accurate results. EEE, on the other hand, has been utilized from the past decade in software estimation to avoid biasness generated by solo ML learners. Recently, there is an increasing trend on metaheuristic optimization (MO) methods in field of SDEE claiming to get more accurate results. This section describes previous work on ensemble estimation and metaheuristic optimization. Summary accumulated from literature is presented next (Section 2.3: Summary of literature work limitations) along with problem formulation of this study (Section 2.4: Problem formulation and research question).

### 2.1. Ensemble effort estimation (EEE)

As discussed earlier, the phenomenon of conclusional instability has initiated a thorough discussion on overcoming inconsistent findings. Among other solutions, the utilization of ensemble approaches seems to attract the interest of SDEE community. Previous SDEE studies have also supported the concept of using ensemble. An SLR conducted by MacDonell and Shepperd [25] on SDEE reported that using a combined prediction coming from multiple models can improve prediction accuracy if no dominant technique is found. This point of view is also verified later by another SDEE research review done by Jorgensen and Shepperd [7] where it is explicitly concluded that aggregated results generated by single models are more accurate than results of individual techniques. These findings are further verified by ensemble effort estimation SLR conducted by Idri et al [6] The systematic review analyzed 24 studies published between 2000 to 2016 in the following aspect: solo learners utilized to construct ensembles, accuracy achieved, combination rule applied, performance achieved by ensembles in comparison with solo models. The principal findings derived from the studies are;

- 17 out of 24 studies used homogeneous ensemble, making them most frequently utilized ensemble technique.
- 16 solo learners are used to construct ensemble models out of which, ML techniques are frequently incorporated.
- For single base learners, 12 studies applied ANNs and DTs.
- Heterogenous ensemble is explore by 9 out of 24 studies, which yielded better performance compared to their base algorithms (with MMRE = 62.48%, Pred(25) = 43.35%, and MdMRE = 26.80%).
- 20 combination rules are utilized to generate final estimation, and more accurate results are achieved via linear combination rule, also referred as arithmetic mean combiner.
- Overall heterogenous ensembles used combination of 12 single techniques among which, DTs and kNN are frequent choices of heterogenous ensemble members.
- Overall, the results defined that ensemble models produced more accurate results, compared to their base leaners.

The SLR projected a thorough analysis on effectiveness of EEE, however no conclusion is found regarding the best EEE techniques. This work also analyzed recent EEE studies to identify commonly utilized ensemble techniques, base algorithm choices, combination rules and preprocessing technique applied.

A summarized view of EEE studies is presented in Table 1, containing base algorithms used in the study, ensemble technique applied, experimental setup along with model being compared to proposed approach and main conclusion derived from that study. The analysis of past EEE studies also brings conclusion on selecting solo ML leaners. The rationale behind choosing three base algorithms (i.e., RF, SVR and DeepNet) for creating ensemble are:

**Table 1:** Summary of EEE studies.

Main Finding	1. No best single classical/fuzzy analogy technique across all datasets. 2. Ensembles of both analogy techniques work better, particularly, fuzzy analogy ensemble outperformed.	Promising improvement in heterogenous ensembles using combination rules rather than solo models. Models with IRWM combination rule is giving higher effect size and SA	No clear assumption on combination rule surpassing all contexts, however ensemble with median combination rule is more successful.	No technique favored by all datasets, but SVR, kNN are most occurring solo learners. 2. GS, PSO optimized models are better than WEKA default models.	1. As point estimator, proposed model outperforms or provided similar performance compared with other methods. 2. As uncertain predictor, model achieved significantly narrower PIs compared to base learner.
Comparing models	1. Solo classical analogy 2. Solo fuzzy analogy 3. Homogeneous ensembles of classical analogy 4. Homogeneous ensemble and fuzzy analogy.	1. kNN, SVR, MLP, M5Prime algorithm (M5P) 2. Ensemble models with three combination rules	1. Models with and without two feature selection methods 2. Ensemble methods with three combination rules	1.Solo techniques optimized with GA, PSO and WEKA default parameters. 2. Ensemble of optimized solo techniques with three combination rules	RVM, Bagged-RVM kNN, SVR, MLP, RT, Bagged-RT, Bagged-SVR
Performance evaluation	1. MAE, MBRE, MIBRE, LSD, SA, Effect-size 2. Kolmogorov Smirnov test for verifying normal distribution of absolute errors (AEs) 3. Box-Cox transformation for AEs	1. Pred, SA, Effect-size 2. Scott-knott (SK) for models statistical difference	1. MAE, MdAE, MIBRE, MdIBRE, MBRE, MdBRE, PRED, LSD 2. Scot-knott (SK) test & Borda count for model ranking	1. MAE, Pred, MIBRE, MBRE, LSD, SA, Effect-size	1. MAE, MdAE, LSD, SA Hit rate Relative width 2. Friedman tests for model performance significance
Experimental Setup	1. Max-min normalization 2. LOOCV 3. Box-Cox transformation	1. Parameter setting with preliminary round of executions having lowest MAE	1. Feature selection with Correlation & RReliefF based 2. Parameter setting with GS 3. LOOCV	1. Parameter setting with GS, PSO	1. Normalization with zero-mean method 2. 10-fold CV 3. Parameter setting wth rid search (GS) 4. Feature selection with one-way ANOVA
Ensemble combination	Average, Median, Inverse ranked weighted mean (IRWM)	Average, Median, Inverse ranked weighted mean (IRWM)	Average, Median, Inverse ranked weighted mean (IRWM)	Average, Median, Inverse ranked weighted mean (IRWM)	Bagging created with: Empirical Mean, Univariant Empirical Probability density functions (PDFs) Bivariant Empirical PDFs
Dataset	1. PROMISE Albrecht COCOMO8 China Desharnais, Kemerer Miyazaki 2. ISBSG	1. PROMISE Albrecht, Miyazaki	1. PROMISE Albrecht, China, COCOMO81, Desharnais, Kemerer, Miyazaki	1. PROMISE Albrecht, COCOMO81, China, Deharnais, Kemerer, Miyazaki, 2. ISBSG R8	1. SEACRAFT Maxwell,, Kitchenham,, Cocomo81, Nasa93 2. ISBSG R8
Base algo	Classical-Analogy, Fuzzy-analogy	kNN, SVR, MLP, M5Prime algorithm (M5P)	kNN, SVR, MLP, DT	kNN, SVR, MLP, DT	Relevance Vector Machines (RVM)
Objective	Investigating effectiveness of analogy-based estimation (ABE) including classical & fuzzy analogy by creating homogeneous ensembles with three combination rules.	Investigating impact of heterogenous ensemble with four ML techniques combined with three combination rules.	Examining effect of two filter feature selection methods (Correlation based and RReliefF feature selection) on ensemble performance	Investigating impact of parameter setting on ensemble members using GS, PSO and WEKA default parameters	Proposing prediction interval (PIs) and confidence levels using bootstrap samples of RVM models and modified training bags replaced by their synthetic counterparts.
Study	[20]	[26]	[27]	[13]	[23]
Main Finding	AdaBoost and Bagging MLP has highest R2 compared to solo MLP and other ensemble models.	Significant of proposed ABC-based ABE is found particularly on ISBSG dataset.	Proposed recursive SVR based feature elimination method outperformed than other comparing models in both training and testing.	1. Accuracy optimized models is higher compared untuned parameters. 2. PSO tunned stacked model performed better than GA.	1. Proposed metaheuristic weighted ensembles of hybrid search-based algorithms performed better than ML and other ensemble models. 2. FFA based optimized weight ensemble has shown best performance overall.
Comparing models	MLP, Ridge-MLP, Lasso-MLP, Bagging-MLP, AdaBoost-MLP	ABE, GA-ABE DE-ABE, PSO-ABE	GA‐ABE PSO‐ABE SADE‐ABE Joint angle and delay estimation (JADE‐ABE) differential evaluation (DABE)	1. Stacked models without any parameter tunning 2. Stacked model with GA and PSO parameter tuning	1. LR, RF, MLP 2. Individual CART_R, FRSBM_R 3. Ensemble with FFA, PSO and GA based optimized weights.
Performance evaluation	R2	1. MMRE, Pred 2. Friedman tests for model performance significance	MMRE, MMER, MBRE, MIBRE, Pred	2. Scot-knott (SK) test & Borda count for model ranking	MAE, MMRE, Pred
Experimental Setup	N/A	1.Normalization with 0–1 method 2. Three-fold-CV	Feature selection with enhanced SVR	1. Parameter setting with GA, PSO 2. Missing Data Treatment (MDT) for removing missing data values 3. Feature selection with Pearson correlation	1. Hold-out cross validation method
Ensemble combination	MLP ensemble created with Ridge, Lasso, Bagging, AdaBoost	Multiple variants of ABE with feature weight adjustment in similarity function	Bagging	Stacking with SVR as meta learner	Ensemble with Optimized weight
Dataset	1. PROMISE Deharnais	1. PROMISE Desharnais, China, Maxwell Nasa93, Cocomo81 2. ISBSG R11	1. PROMISE COCOMO NASA93	1. ISBSG	1.PROMISE Desharnais, COCOMO, NASA93
Base algo	MLP	kNN SVR MLP DT	RF	Linear regression (LR), MLP, RF, AdaBoost	Classification & regression Tree (CART_R), Fuzzy & random sets-based modeling (FRSBM_R)
Objective	Exploring MLP with its homogeneous ensemble versions.	Investigating ensemble of analogy-based estimation (ABE) with inclusion of artificial bee colony (ABC).	To predict the cost, effort, size and schedule consistently by extracting relevant features and eliminating weakest features with recursive SVR.	Examining impact of parameter setting with GA, PSO on performance of stacking ensemble	Determining performance efficiency of hybrid search-based algorithms along with optimized ensemble weights using FFA, PSO, GA
Study	[28]	[29]	[30]	[3]	[31]

- Tree-based models (DTs) are mostly used as stated by SLR of [11] RF is also bagging form of DTs hence we considered this as a constituent of ensemble creation.
- According to SLR on EEE conducted by Idri et al [6], most ensemble studies found, have selected ANNs as solo learners, hence DeepNet is decided to be part of ensemble in the study.
- In Table 1, there is a trend of using models like RF, SVR and Neural network in most EEE studies as base algorithms.

All these conclusions bring us to use these three algorithms as solo leaners to carry out our proposed ensemble scheme.

### 2.2. Metaheuristic optimization (MO)

Parameter setting is a significant criterion for a single ML estimation technique to perform well in predicting effort. Training ML learner requires optimal set of hyperparameters, depending upon dataset and problem targeted. However, setting optimal hyperparameters is not trivial. Manual hyperparameter setting is done by trial and error or other search-based methods as grid search or random search [32]. Manual hyperparameter tuning requires a lot of overhead and become exhausting when parameter search space is large. For that reason, automatic hyperparameter tuning is being considered. Metaheuristic optimization is getting recognition in the field of ML hyperparameter tuning to increase algorithm’s efficiency. Metaheuristic algorithms are simple, highly parallelizable and work best on hyperparameter space [33, 34].

In past SDEE studies, metaheuristic algorithms contributed in providing optimized solution for hyperparameter of single ML technique. However, MO is not included for hyperparameters only. Few researchers included MO in EEE for optimizing weights of various individual models to get best estimation results. Most widely used MO in effort estimation domain include, particle swarm optimization (PSO), genetic algorithm (GA) [35] firefly algorithm (FFA) [36] and bat algorithm (BA), Artificial bee colony (ABC) [37, 38]. Table 2 shows previous EEE work particularly incorporated metaheuristic algorithms for hyperparameter optimization or optimized weight learning for ensemble.

**Table 2:** Metaheuristic optimization work.

Study	GS	GA	PSO	BH	ABC	FFA	BA
[29]					†		
[27]	√						
[23]	√						
[39]	√						
[40]						†	†
[41]	√						
[3]		√	√				
[13]	√		√				
[31]		†		†		†	
[42]		√†					

Hyperparameter optimization (√)

Ensemble weight optimization (†)

### 2.3. Summary of literature work limitations

From the literature work discussed above, it is possible conclude:

- Utilization of both accuracy and diversity measure simultaneously are not well-formulated. A little to no attempt is made to assess the impact of two significant criteria i.e., accuracy and diversity in ensemble’s working.
- Although, MO is considered for achieving optimal hyperparameter for single ML, but utilizing MO in both perspectives, i.e., for hyperparameter optimization and weight optimization of ensemble is not considered under one framework. Although work of [42] applied the use of GA for optimizing parameters of analogy technique and then determining weights for ensemble creation, but ensemble constituents are homogeneous versions of analogy-based techniques. Ensemble weights optimization of diverse ML algorithms to create ensemble is still not incorporated.
- Hyperparameter optimization of ML algorithms entails search space to get best configuration of hyperparameters by search-based or metaheuristic algorithm. For a particular optimization problem, criteria for defining and selecting search space is not part of any SDEE work.
- Although ample amount of work is done in the field of EEE. Researches have fairly incorporated homogeneous and heterogenous ensemble in developing SDEE model but power of using both kinds of ensemble simultaneously is not explored Publicly available effort estimation repositories contain heterogenous datasets, differing in software projects size, field, organization and dissimilar effort drivers presented in each dataset. Homogeneous ensemble may give acceptable results on one configuration of dataset and fail to estimate effort for a different setting, while heterogenous is suitable in those conditions. For that reason, if both types of ensembles are present in one model, then it would bridge the gap of dataset and configuration uncertainties.

### 2.4. Problem formulation and research questions

Limitations presented in previous literature (Section 2.3: Summary of literature work limitations) are considered for problem formulation of this wok listed below:

- ***Problem*:** Absence of accuracy and diversity considerations while creating ensemble.
  ***Solution*:** This work incorporates both measures by utilizing multi-dimensional bagging (diversity) and MO (accuracy).
- ***Problem*:** Analyzing the impact of both optimization domains (hyperparameter optimization and optimal weights assignment) while creating ensemble is missing.
  ***Solution*:** Optimization of both perspectives I spart of this study. Hyperparameters of base algorithms are optimized (using MO), then combined in heterogenous ensemble with optimized weights (optimized with MO).
- ***Problem***: For ML hyperparameter optimization, no consideration is made on defining search-space selection criteria.
  ***Solution*:** This work proposed to include search-space division, for getting optimized hyperparameters from multiple sub-sections of same larger search-space.
- ***Problem*:** Investigating the use of both ensemble mechanisms (Homogeneous and Heterogenous) simultaneously is overlooked.
  ***Solution***: This work contributes in combining both kinds of ensemble, i.e. homogeneous (in form of multi-dimensional bagging) and heterogenous (in form of Metaheuristic- optimized weighted ensemble).

From this perspective, this study attempts to address following research questions:

***RQ1*: Does optimization included in both domains (hyperparameter optimization and optimal weights assignment) improve estimation performance?**
***RQ2*: Does performing search space division endorse same results as utilizing entire search space?**
***RQ3*: Does integration of Homogeneous and Heterogenous ensemble tend to improve the performance or Homogeneous/Heterogenous ensemble alone can give good performance?**

## 3. Background

This section elaborates the details of techniques used in this study, including solo ML algorithms and optimization technique.

### 3.1. Random Forest (RF)

RF incorporates bagging techniques, referred to as “bootstrap aggregation”. This involves generating a new dataset from an existing one through bootstrap sampling with replacement [43]. RF implementation involves two major strategies: (1) Forming a certain number of trees with data using different bootstrap samples (i.e., bootstrap with replacement). (2) For splitting each node, randomly chosen best feature among the “subset of predictors” is used, instead of taking best split among all variable for splitting each node, like standard trees [44–46]. This study implements RF working as follows:

- *Random bootstrap sampling*: Random bootstrap sampling is performed with replacement for each dataset D, containing d observations, and F features. These bootstrap samples are then used for training ‘*n’* number of base trees.
- *Random Feature Selection*: For the n^th^ base tree, a subset of m features is randomly selected for model training. Constant tree structure Ͳ^n^_m_ is learned for nth base tree. For node splitting, instead of traversing every possible split in all F features, Ͳ^n^_m_ only consider splits in a randomly selected feature subset.
- *Pruning*: Pruning operation is performed on constructed constant tree Ͳ^n^_m_ with M5 based method. After pruning, linear decision tree model ƒ_m_ (x) is formed by converting Ͳ^n^_m_ tree structure. For final pruned decision tree, having P leaf nodes, regression function for p^th^ leaf node is denoted by Һ_p_ (x). Prediction generated from entire decision tree model is presented by Eq 1, where *ℊ*(*a*) is a conditional function which returns 1 if *a* is true and returns 0 otherwise.
$${\text{f}}_{\text{m}}(\text{x})=\sum_{p=1}^{P}ℊ\left(x\in X_{p}\right).{\text{h}}_{\text{p}}(\text{x})$$ (Eq 1)
- *Average aggregation*: Randomized decision trees are constructed in parallel with above-mentioned process, resulting N base trees. For a sample x, prediction from N base trees is generated. Final estimation (*ℱ* (x)) is made by averaging the prediction of N base models (ƒ_m_ (x)) mentioned in Eq 2.
$$F(\text{x})=\frac{1}{N}\sum_{n=1}^{N}{\text{f}}_{\text{m}}(\text{x})$$ (Eq 2)

Since regression trees has tendency to overfit for small datasets, which in-turn considerably effects performance of RF. To avoid this, there is a need of hyperparameters optimization for RF for better performance. Hyperparameters for RF include: the number of trees constituting the forest (*ntree*), the number of features randomly selected at level of each node (*mtry*), minimum number of data samples in a leaf node (*nodesize*) and the size of in-bag samples (sampsize).

### 3.2. Support vector Regression (SVR)

SVR is among most frequently utilized ML technique in SDEE domain, suitable for linear/nonlinear regression. [47–50]. Regression based on SVR consist of data $\text{D}=\left\{{\text{a}}_{1}^{\text{n}},{\text{b}}_{1}^{\text{n}}\right\}$ where a_i_ is vector of independent variables, while b_i_ corresponding scalar real dependent variable. SVR regression equation in feature space can be represented as Eq 3:

Y(a,w)=(w.Φ(a)+c) (Eq 3)

where, w defines the weight vector, c is a constant, Φ(a) is the feature function while w.Φ(a) represents dot product of two terms. Lagrangian multiplier *β* and β^*^ are also incorporated and only non-zero coefficients, along with their input vectors, a_i_, are termed the support vectors. The final form comes out as represented in Eq 4:

$$\left.\text{Y}\left(\text{a},\beta ,\beta^{*}\right)=\sum_{i=1}^{n}\left(\beta_{\text{i}}-\beta_{\text{i}}^{*}\right)\left(\Phi \left({\text{a}}_{\text{i}}\right)\cdot \Phi \left({\text{a}}_{\text{j}}\right)\right)+\text{c}\right)$$ (Eq 4)

By the help of kernel function K(xi,xj), the SVR function can be obtained by Eq 5 as given below:

$$\left.\text{Y}\left(\text{a},\beta ,\beta^{*}\right)=\sum_{i=1}^{n}\left(\beta_{i}-\beta_{i}^{*}\right)K\left(\text{a},{\text{a}}_{\text{i}}\right)+\text{c}\right)$$ (Eq 5)

Kernal plays an important part in SVR implementation. Radial basis function (RBF) kernels are the most generalized form of kernelization and is one of the most widely used kernels due to its similarity to the Gaussian distribution. The RBF kernel function for two points xi,xj computes the similarity between them or how close they are to each other. RBF can be mathematically represented by Eq 6 as follows:

$$\text{K}(\text{xi},\text{xj})=\exp\left(-\gamma ||{\text{x}}_{\text{i}}-{\text{x}}_{\text{j}}|{|}^{2}\right)$$ (Eq 6)

||x_i_ − x_j_|| is Euclidean (L2-norm) distance between two points x_i_,x_j_. In RBF, *γ* is parameter used to tune the equation, and computed as follows in Eq 7, where *σ* is the variance.


$$\gamma =\frac{1}{2\sigma^{2}}$$ (Eq 7)

The key parameters need adjustment for SVR are; complexity or penalty parameter C (also referred as *cost*) which controls the trade-off between error minimization and margin maximization. Its optimization is necessary as to have an idea how much misclassifying must be avoided on each training sample. Another parameter of SVR, *epsilon* (ε), defines the extent to which deviations (i.e., errors) are tolerated. For model training, SVR works by finding a function with at most ε deviation from actual value of dependent variable in all data samples, while keeping function as flat as possible. *epsilon* (ε) optimization is required since, function to adjust tolerable error of the regression model is essential. Another optimizable parameter, *gamma* (γ) suitable for radial basis function (RBF) kernel [47]. *gamma* parameter defines how far the influence of a single training example reaches.

### 3.3. Deep neural network (DeepNet)

Deep learning is quite potential ML-SDEE technique, exploiting precision abilities of Deep Neural Network (DeepNet) [51]. The reason is its ability to represent complex relationships between dependent variable (effort) and independent variables (effort drivers). For building effort estimation model, DeepNet is construed using three basic layers. (a) An input layer containing input neurons (effort drivers); (b) hidden layers with neurons that calculate their output by means of an activation function. Let L denotes hidden layer, for modeling DeepNet, the number of hidden layers would be L > 2 (c) an output layer which takes output from hidden layer neurons and accumulates linear weighted sum, serves as prediction of network (i.e., estimated effort). Since effort estimation is regression problem, so in case of regression DeepNet has only one output neuron. The layer labeled X is the input layer which contains all the explanatory variables k in the data set. The layers labeled Z are the hidden layers. The number of hidden layers L can be arbitrarily set, however, in this study, three hidden layers are used. Each hidden layer can contain arbitrarily many neurons denoted by *vl* where *l* stands for the *l*^*th*^ hidden layer. Z is a vector of outputs from all neurons of the (*l* − 1)^*th*^ layer represented as follows in Eq 8

$$Z_{l-1}=\left(z_{l-1,1},z_{l-2,2},\cdots ,z_{l-1,v-1}\right)$$ (Eq 8)

The output *z*_*lv*_ of the *v*^*th*^ neuron in the *l*^*th*^ hidden layer is represented in Eq 9:

$$z_{lv}=f\left(w_{0lv}+w_{lv}^{\prime }Z_{l-1}\right)$$ (Eq 9)

The function *f*(*a*) is referred as the activation function. Sigmoid is commonly used activation function and apart from Sigmoid, Rectified Linear Unit (ReLu) is also a popular choice and considered appropriate for the reduction of overfitting influence [52]. Sigmoid and ReLu are shown in Eqs 10 and 11.


ReLUx=max0x (Eq 10)

$$Sigmoidx=1+e^{-x^{-1}}$$ (Eq 11)

The outputs of the first, hidden, and output layers are expressed as Eqs 12 and 13 and 14.

$$h_{i}=\sigma W_{i}^{v}x+b_{i}$$ (Eq 12)

$$h_{l}=\sigma W_{l}^{v}h_{l-1}+b_{l}$$ (Eq 13)

$$\hat{y}=W_{o}^{v}h_{l}+b_{o}$$ (Eq 14)

where *W* and *b* represent the weight matrix and bias vector of the l^th^ hidden layer, respectively. For the input layer, the vector of explanatory variables of input features (*x*) is used while for hidden layers, computed values of previous hidden layer (*h*_*l*-1_) is used.

Performance of DeepNet is largely dependent on optimal hyperparameter configuration including: (1) number of hidden units (OR *size* of DeepNet) (2) *decay* (3) the number of epochs used in training (4) the learning rate and (5) the momentum. In this study, parameters selected for optimization are number of hidden neurons *size* and *decay*.*size* parameter can affect DeepNet’s performance since depth of hidden layers enhance data fitting capability of DeepNet model. Less hidden layer size causes underfitting, making model to inadequately detect the signals in a complicated data set. While increased hidden neurons may result in overfitting and unnecessary model complexity, i.e., model will have larger information processing capacity with little information to be processed on. Hence an optimized value of hidden layer neuron is required for better functioning DeepNet. Other parameter, *decay*, is regularization parameter preferably to work with L2 regularization to avoid over-fitting. It is used to keep the weights small to avoid oversizing the gradient. For a regression problem, L2 regularization is suggested to apply [53]. L2 norm of weights are added to the loss, which might result in loss getting quite large. Due to this, DeepNet model tend to set all model parameters to 0. Hence, optimized value of *decay* is required, to keep weights small and preventing weights to grow out of control. Also, optimization of *decay* is needed because, with too much weight *decay* model never quite fits and too little weight *decay* causes model to stop a little bit early.

### 3.4. Firefly Algorithm (FFA)

As discussed earlier, performance of ML technique largely correlates to accurate parameter setting. Moreover, hyperparameter tunning differs from dataset and the application context [23, 54]. Hence, for a given ML technique, using the same parameters for multiple datasets can influence its prediction ability. Therefore, this work put focus on getting optimal hyperparameters setting for each ML algorithm with respect to the dataset. Moreover, for heterogenous ensemble, weights assigned to each model’s prediction is also critical to be optimized, for getting high performing ensemble. A metaheuristic is a search-based heuristic, for getting best solution of optimization problems having partial scope with limited information present [31, 55]. MO provides optimal or even sub-optimal solutions for highly nonlinear and multimodal optimizations problems.

FFA falls under the category of bio-inspired algorithms, i.e., inspired from the behavior of the swarm such as bird folks, insects, fish schooling in nature. FFA was originally proposed by Xin-She Yang [56] and serves well in metaheuristic optimization. According to some recent studies, FFA evolves as promising algorithm as it outperforms other metaheuristics such as genetic algorithm [57].

FFA is based on population of fireflies and works by adapts their rhythmic flashing light behavior for attracting mating partners or potential preys. The light intensity of a firefly lessens when it moves away from other firefly and modifies according to inverse square law. The light intensity (brightness) of firefly (referred as L) decreases when distance h from its source increases, i.e., L ∝ 1 /h^2^. Also, L decreases by light absorbed in the environment. This behavior contributes in moving from a locally optimal solution to a globally optimal solution. Rules of FFA suggest that all fireflies are unisex, so attractiveness criteria between two fireflies is “brightness” of each firefly. Brighter firefly attracts less bright firefly, hence less bright firefly moves towards brighter one. However, for a firefly, if there is no brighter firefly, it will perform random walk i.e., move randomly. Brightness L is directly related with the objective function. For a particular position p, the brightness of firefly will be is chosen as L(p) ∝ f(p), where f(p) is objective function in optimization problem which needs to be maximized or minimized. The combined effect of both, the inverse square law and absorption, can be approximated to Gaussian form as *L(h) = L*_*0*_*e* ^*−γh2*^. Since a firefly’s attractiveness is proportional to the light intensity, the attractiveness function of the firefly can be defined as; *β(h) = β*_*0*_*e* ^*−γh2*^; where β0 is the attractiveness at h = 0 and γ is the light absorption coefficient

Attractiveness of firefly depends on its brightness, which varies according to distance between two fireflies. So, for two fireflies, firefly *i* and firefly *j*, attractiveness β will be less if distance h_ij_ between them is greater. Cartesian distance is taken between any two fireflies i and j located at positions p_i_ and p_j_ respectively according to Eq 15 [58].


$$h_{i,j}=||p_{i}-p_{j}||=\sqrt{\sum_{r=1}^{m}{\left(p_{i,r}-p_{j,r}\right)}^{2}}$$ (Eq 15)

Where *p*_*i*,*r*_ is spatial coordinate *p*_*i*_ of *ith* firefly. If fitness of firefly *i* is greater than fitness of firefly *j*, then *i* will move from a current position p_i_ towards the position p_j_ (of firefly *j)*, and movement is determined by Eq 16.


$$p_{i}=p_{i}+\beta_{o}e^{-\gamma h_{ij}^{2}}\left(p_{i}-p_{j}\right)+\alpha \left(rand-\frac{1}{2}\right)$$ (Eq 16)

Where *p*_*i*_ is the current position of a firefly, the second term i.e., $\beta_{o}e^{-\gamma h_{ij}^{2}}\left(p_{i}-p_{j}\right)$ is the firefly attractiveness with respect to light intensity (L) observed by nearby fireflies. Third term *α*(*rand*– 1/2) is the random walk of firefly when there is no brighter firefly; where *α* is a randomization coefficient and *rand* is a random number, both can be drawn from normal distribution.

## 4. Methodology

This section describes working of proposed effort estimation scheme. Proposed framework applies EEE and combines both types of ensemble methods i.e., Homogeneous and Heterogenous ensemble.

**Fig 2:**
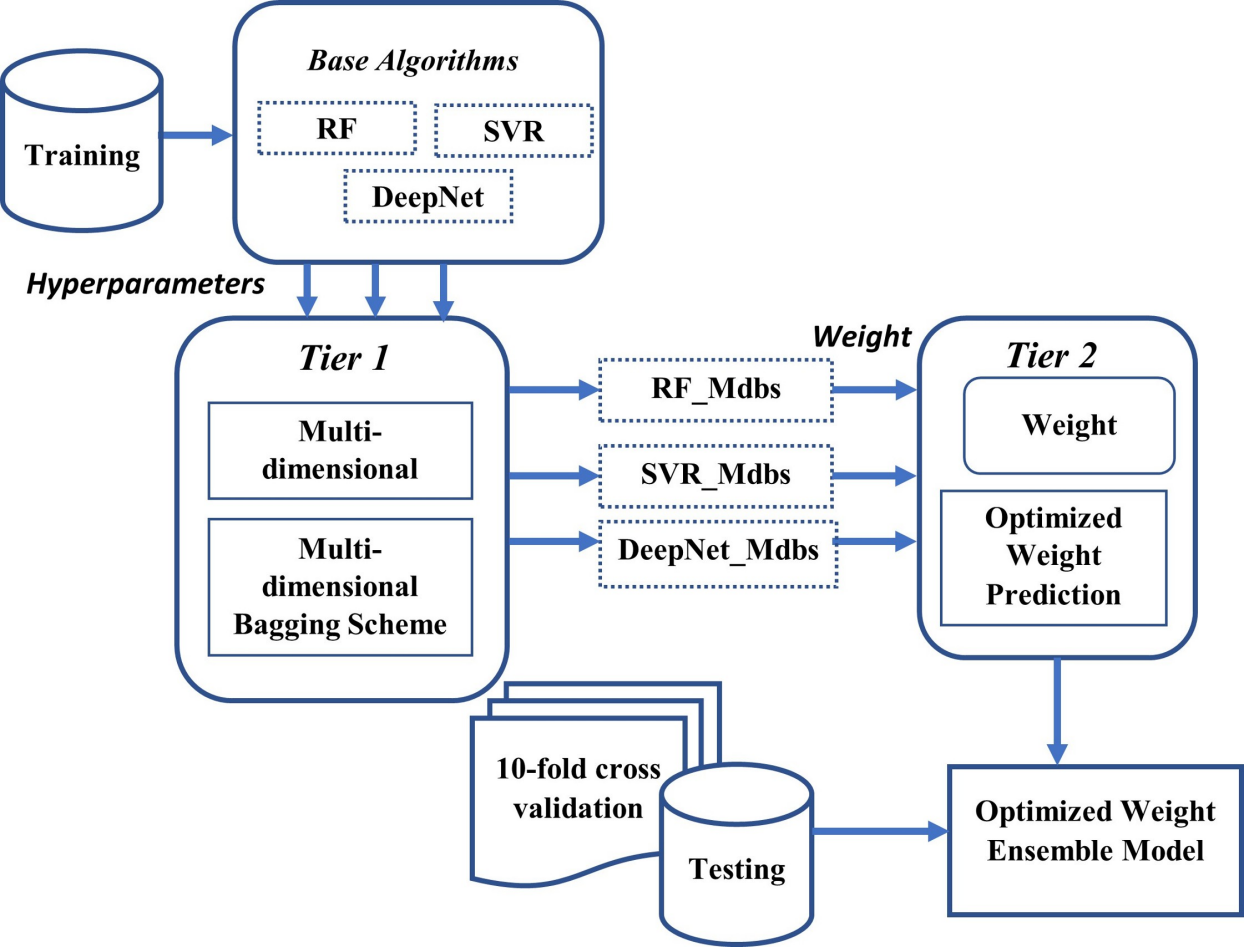
Proposed MoMdbWE framework.

Homogeneous ensemble part focuses on creating maximum possible diverse bags of base algorithm by the use of proposed *Multi-dimensional bagging (Mdb)* technique. For accuracy achievement, use of MO algorithm is included to get best hyperparameters of base algorithms before combining them into ensemble. Heterogenous ensemble part focuses on combining Mdb scheme of each base algorithm, with their optimized weights again obtained from metaheuristic algorithm. Final estimation is made by ensembling each Mdb scheme (of base algorithm) with their optimized weights. The general view of proposed scheme is shown in Fig 2. Proposed framework is implemented in two tiers. First tier (Tier-1) works on creating Multi-dimensional bagging (Mdb) scheme and second tier (Tier-2) works on creating *Metaheuristic-optimized weighted ensemble (MoWE)*, both together referred as **M**etaheuristic **O**ptimized **M**ulti-**d**imensional **b**agging and **W**eighted **E**nsemble (MoMdbWE). Details of each tier is elaborated in Section 4.1: Tier-1: Multi-dimensional Bagging (Mdb) Scheme and Section 4.2.: Tier-2: Metaheuristic-optimized Weighted Ensemble (MoWE)

### 4.1. Tier-1: Multi-dimensional Bagging (Mdb) scheme

Tier-1 works by taking individual base algorithm (solo learners) at a time and creating multi-dimensional bagging scheme for each base algorithm. Base algorithms used in this study are RF, SVR, DeepNet.

**Fig 3:**
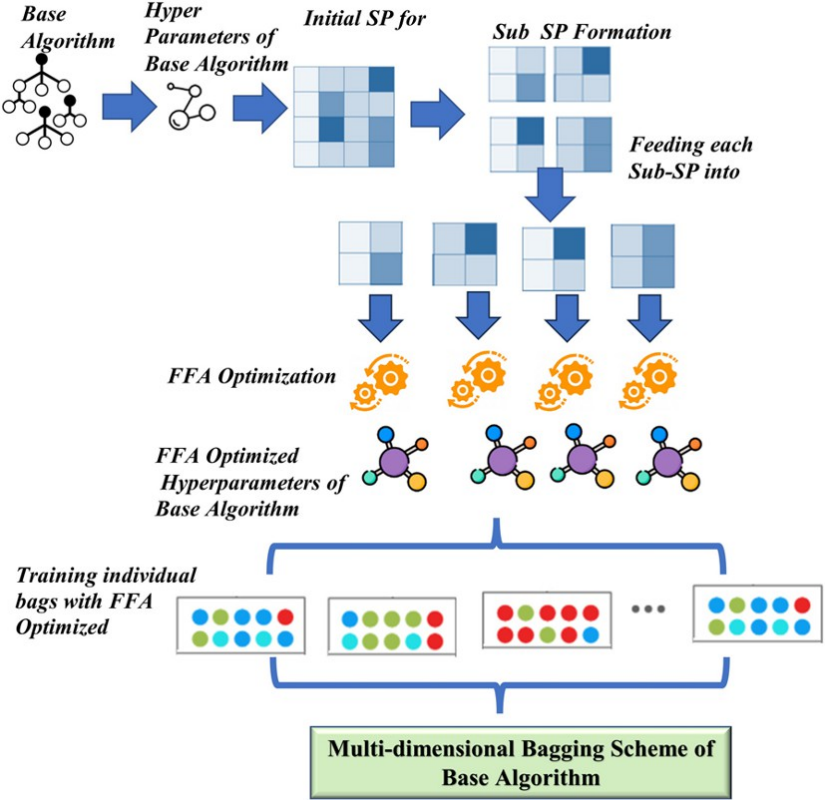
Tier 1-Multi-dimensional Bagging (Mdb) scheme.

For one particular base algorithm, initial search space (SP) of its hyperparameters is defined. Initial search space (SP) of hyperparameters is then divided using proposed multi-dimensional search sub-spaces (Sub_SPs) formation technique. Number of dimensions in initial search space (and in Multi-dimensional bagging) depends on number of hyperparameters to be optimized for single base algorithm i.e., for a base algorithms with *m* optimizable hyperparameters, *m-dimensional* SP and Sub-SPs will be formed for creating Mdb. Best hyperparameters from each Sub_SP are then obtained with the use of MO. In this study Firefly algorithm (FFA) [40] is used for optimization due to its promising results on tuning hyperparameter of three base algorithms (Appendix A: Table 13 in S1 File). Each Sub_SP gives one set of optimized hyperparameters. These N sets of optimized hyperparameters (coming out of N Sub_SPs) are then used to train N number of bags (bootstrap samples with replacement) from the dataset. These N-bags, trained from optimized hyperparameters are then combined in the form of bagging to get *Multi-dimensional bagging (Mdb)* Scheme for one base algorithm. Same process is repeated for remaining base algorithms to get Mdb scheme for each base algorithm. Fig 3 depicts complete working process of Tier-1.

Steps for implementing Mdb scheme is as follows:

1. Take one base algorithm *Z* and select its hyperparameters to be optimized.
2. For m-hyperparameters, create m-dimensional grid. This m-dimensional grid will act as initial SP for algorithm *Z*.
3. Create N Sub_SPs by dividing the Initial SP (m-dimensional grid) according to Algorithm 1.
4. Feed each Sub_SP separately, into firefly algorithm (as range with lower bound and upper bound).
5. For N Sub_SPs, obtain N sets of optimized hyperparameters, according to Algorithm 2.
6. Create N bootstrap samples (with replacement) from the dataset. Use one *sample*_*n*_ to train algorithm Z with one set of optimized hyperparameter coming from steps 4–5. This will produce one bag for Algorithm Z.
7. Repeat the same process for remaining N-1 bootstrap samples and create total N-bags for algorithm Z.
8. Combine all N trained bags to have *Multi-dimensional bagging (Mdb)* scheme for algorithm Z.
9. Repeat step 1 to 8 for remaining base algorithms.

***Algorithm 1: Sub-Search spaces (Sub_SPs) formation from initial* SP**
***Input*:** Algorithm (Z); where [Z} ϵ [RF, SVM, DeepNet}
m = number of hyperparameters of Algorithm (Z) and highest dimension of grid
N = No. of Multi-dimensional bags to be formed;
error = error value point (MAE) in m-dimensional grid
***Output*:** N search sub-spaces

***Begin*:**

*1. Select Algorithm (Z)*

*2. Select m-hyperparameters of Algorithm (Z)*

*3*.*Creat m-dimensional grid for Algorithm (Z); where m = number of hyperparameters of Algorithm (Z)*
          Algorithm(Z)_Grid [m]

*4. Define N error ranges*

          split = Min_error + Max_error / N
    *for i = 1 upto N*
  *do*
          error_range(i) = split + error_range(i-1)
    *end for*
*5. Create N Sub_SPs [lower bound*, *upper bound]*
        Sub_SP (n) = [(Lower_Bound (n)), (Upper_Bound (n)) ]
    *for j = 2 upto N*
  *do*
     Sub_SP (1) = [0, error_range(1)]
    Sub_SP (j) = [(Algorithm(Z)_Grid(error) > error_range(j-1)), (Algorithm(Z)_Grid(err) < error_range(j)) ]
    *end for*
***Return*** N Sub_SPs

***End***

***Algorithm 2: Firefly Algorithm for Optimized Hyperparameter of N Sub_SPs***
***Input***: m = No. of hyperparameters to be optimized for base Algorithm
max_pop = Maximum population of fireflies
I_o_ = initial light intensity at h_0_; γ = Light absorption coefficient; h = Distance between two fireflies;
t = iteration; α = randomization parameter; δ_i_^t^ = vector of the random number drawn from Gaussian distribution at iteration t; β0 = Attractiveness at h = 0; α ∈ [0,1]; rand ∈ [0,1]; scale = (Lower_bound- Upper_bound)/Upper_bound
***Output*:** Best firefly (optimal solution) for each N Sub_SP

***Begin***

1. Assign j^th^ Sub_SP as range for FFA
        *for each* Sub_SP *j = 1 upto N*
  *do*
          Lower(Sub_SP (j)) = Lower_bound
          Upper(Sub_SP (j)) = Upper_bound
*2. Initialization random population of Fireflies;* Xj; where j = {1,2, …, max_pop} and Xj = {x_j_1, x_j_2 … x_j_m}
     $Xj=\;Lower_{bound}+\;rand.*\;\left(Upper_{bound}-Lower_{bound}\right)$ (*Eq (a))*
*3. Calculate distance between firefly (Xj) and firefly (Xk) a*cc*ording to Eq(b)*
  $h_{j,k}=∥Xj-Xk∥=\sqrt{\sum_{r=1}^{m}{\left(Xj,r-Xk,r\right)}^{2}}$ (*Eq (b))*

*4. Calculate the fitness (objective function) of each firefly*

    fit_Algorithm(Xj) {
        return fitness = min MAE }
5. *Update position of firefly*
    *for t = 1 upto max_iteration*
  *do*
    *If* t = max_iteration,
    *then* return X_j_^t^ as Xj(best); return the best position as an optimal solution
    *else if* t ≠ max_iteration and
          fit_Algorithm(Xj) < fit_Algorithm(Xk); firefly(Xj) moving to more attractive firefly(Xk)
        *then* move position of firefly(Xj) toward firefly(Xk) *a*cc*ording to Eq(c)*
     ${\text{X}}_{\text{j}}^{\text{t+1}}{\text{=X}}_{\text{j}}^{\text{t}}{+\beta \text{o}\;\text{e}}^{{-\gamma \text{h}}_{\text{jk}}^{\text{2}}}\left({\text{X}}_{\text{j}}^{\text{t}}{-\text{X}}_{\text{k}}^{\text{t}}\right){+\alpha \;\delta }_{\text{j}}^{\text{t}}\left(\text{rand}-\frac{\text{1}}{\text{2}}\right)\;*\;\text{scale}$ (*Eq (c))*
          *else if* no brighter firefly than firefly (Xj) according to *Eq(d)*
          *then* perform random walk
    ${\text{X}}_{\text{j}}^{\text{t}+1}=\alpha \delta_{\text{j}}^{\text{t}}\left(\text{rand}-\frac{1}{2}\right)*\;\text{scale}$ (*Eq (d))*
          *end if*
6. *Determine fitness of firefly on updated position*
          *C*al*c*ulate fit_Algorithm (X_j_^t+1^)
       *If* fit_Algorithm (X_j_^t+1^) < fit_Algorithm (X_j_^t^)
       *then* Update
         firefly(X_j_^t^) with firefly(X_j_^t+1^)
    *end if*
    return firefly (X_j_(best))
    where X_j_(best) has min(fit_Algorithm(X_j_))
          *end for each*
**Return** Xj(best) for each Sub_SP (j); j {1, 2, …, N]

***End***

### 4.2. Tier-2: Metaheuristic-optimized Weighted Ensemble (MoWE)

Tier-2 works on combining the output coming from Tier-1 using *Metaheuristic-optimized weighted ensemble (MoWE)*. At the end of Tier-1, three Mdb schemes (one Mdb for one base algorithm) are obtained. Weights for individual Mdb are learned from FFA and weighted ensemble is created. Effort coming from this *MoWE* serves as final effort prediction for a particular dataset. Steps for creating *MoWE* are as follows (Algorithm 3):

1. Divide entire dataset D into training and testing data using 70:30 ratio.
2. For training dataset, train three Mdb schemes separately (obtained from Tier-1).
3. Acquire predictions from each Mdb scheme on training dataset.
4. Obtain weights of each Mdb prediction using Firefly algorithm with minimized MAE
  (i.e. fitness = minimum MAE)
5. Repeat step 2 and 3 for 30% remaining test data and obtain test data predictions.
6. Make final prediction on testing data by creating *MoWE*, i.e., assign optimal weights (obtained from FFA in step 4) to predictions obtained in step 5.

***Algorithm 3*: *Metaheuristic-optimized weighted ensemble (MoWE) creation***
***Input*:** Multi-dimensional bagging schemes for all three base algorithms = {Mdb_Scheme] ϵ {RF_mdb, SVM_mdb, DeepNet_mdb]; w_n_ = weight to be optimized
max_pop = Maximum population of fireflies; t = iteration
***Output*:** Metaheuristic-optimized weighted ensemble (MoWE)

***Begin*:**

*1. Divide dataset D*^*m*^ *into {D*^*m*^_*train*_, *D*^*m*^_*test*_ *] by {70*:*30] ratio*

*2. Define fitness function for FFA*

        fit_weights (w1, w2, w3) {
        return fitness = min MAE ]

*3. Define search space for weights*

        weights search space = w1 → [0.1, 0.9], w2 → [0.1, 0.9], w3 → [0.1, 0.9]

*4. Start FFA*

    for each t = 1 upto max_iteration
        for j = 1 upto max_pop

**On training data, D** ^**m**^_**train**_

*5. Perform training on D*^*m*^_*train*_ *using {mdb_Scheme]*
        model1 = train (RF_mdb, D^m^_train_)
        model2 = train (SVM_mdb, D^m^_train_)
        model3 = train (DeepNet_mdb, D^m^_train_)

*6. Get prediction of above trained models on D* ^*m*^_*train*_

        pred1 = prediction (model1, D^m^_train_)
        pred2 = prediction (model2, D^m^_train_)
        pred3 = prediction (model3, D^m^_train_)
        ensemble_prediction = (pred1*w1) + (pred2*w2) + (pred3*w3)
   return fitness = min MAE
        *end for*
   *end for each*

*7. Obtain best values of ensemble weights from FFA*

        return w1_best_, w2_best_, w3_best_

**On test data, D** ^**m**^_**test**_

*8. Repeat Step 5 and perform training on D* ^*m*^_*test*_

        return model1, model2, model3

*9. Repeat Step 6 and obtain predictions on D* ^*m*^_*test*_

        return pred1, pred2, pred3
        optimized_ensemble_prediction = (pred1*w1_best_) + (pred2*w2_best_) + (pred3*w3_best_)
        return optimized_ensemble_prediction

***End***

## 5. Experimental setup

This section describes the experimental setup for implementation and evaluation of proposed scheme. Since data is not normally distributed as confirmed in Section 5.2: Datasets so max-min normalization rule is applied on all datasets. For validation, 10-fold cross validation method (10-fold CV) is applied for all experiments performed. Rational behind using 10-fold CV as validation method is, since datasets used for implementation are relatively small, hence k = 10 is sufficiently large to avoid the bias created by small training dataset. On the other hand, using k too high (i.e., in leave-one-out where k equal to the size of data) may induce high variance [59]. Each experiment is repeated 5-times with different samples in each run. Performance metrics and statistical evaluation method selected for this study are mentioned in Section 5.4: Performance evaluation measures and Section 5.5: Statistical evaluation. Base algorithms and metaheuristic algorithms are implemented in RStudio, using R programming language. Performance evaluation and statistical tests and other simulation is also performed in RStudio.

For three base algorithms RF, SVR and DeepNet, implementation settings and hyperparameters are mentioned in Table 3. Parameters in bold are the ones which are being optimized by FFA. For SVR implementation, Radial basis function (RBF) is chosen due to its generalization capability, handling space complexity problem and non-linear data [60]. DeepNet is implemented with 3 hidden layers, as complexity level of selected effort estimation datasets is moderate. Choice of activation function in DeepNet is Rectified Linear Unit (ReLu) [52] which is considered appropriate for the reduction of overfitting influence.

**Table 3:** Implementation settings of Base algorithms.

Base Algo.	Implementation setting
RF	***mtry*, *ntree*, *modesize***, trees without pruning
SVR	***cost*, *coef*0, *gamma*, *epsilon*,** Kernel; RBF
DeepNet	***size*, *decay***, No. of hidden layers: 3 Activation function; Rectified Linear Unit (ReLu) Learning rate; 0.01, Regularization; L2, Epochs; 500

Hyperparameters of each base algorithm (Table 4) are then optimized using firefly algorithm (FFA) according to Algorithm 2. For verifying effectiveness of FFA, hyperparameters of base algorithms are also optimized using three other renowned optimization algorithms namely Grid search (GS), GA and PSO. Fitness criteria of all optimization algorithms is taken as minimum MAE. Parameter’s initialization of FFA and compared algorithms (GA, PSO) in shown in, where maximum population size and number of iterations are kept same for all three optimization algorithms to get similar optimization impact [3, 40, 61]. Stopping criteria applied for each optimization algorithm is; when it reaches maximum iteration (i.e., 100) or difference in fitness between one iteration and previous iteration is less than 0.5 [62]. Results of optimization algorithms on all datasets (Appendix A: Table 13 in S1 File), show that FFA provided good hyperparameter optimization for achieving minimum MAE compared to GS, GA and PSO for all three base algorithms. This brough further motivation to use FFA for creating Mdb schemes of base algorithms by optimizing their Sub_SPs.

**Table 4:** Parameters initialization for optimization algorithms.

GA	PSO	FFA
Elitism = 2 Crossover = 0.8 Mutation = 0.1	Local acceleration co-efficient (c1) = 1.5 Global acceleration co-efficient (c2) = 1.5 Inertia weight (w) = 0.7	B0 = 1 Light absorption coefficient (γ) = 1 Randomization parameter(α) = 0.2
Max Iterations = 100	Max Iterations = 100	Max Iterations = 100

Termination condition = reaching 100 iterations

OR MAE (iteration i)–MAE (iteration i-1) < 0.5

For implementing Tier-1, initial SP for each base algorithm is shown in, which is used to form m-dimensional grid (m = number of hyperparameters to be optimized for each base algorithm). This m-dimensional grid is further used for Sub_SP creation according to Algorithm 1. For RF, hyperparameters named *mtry* and *nodesize* will be different for each dataset as they are dependent on number of features and number of samples in each dataset. F is total number of features available in a particular dataset, hence we kept *mtry* as F-1, because last feature of each dataset is output variable i.e., “effort”. The reason to keep all features in initial SP is to explore vast feature range, and it would also give a good division for further Sub_SP formation. Similarly, for *nodesize*, all data samples (projects) in each dataset are considered for constructing individual trees in RF and to have a large margin in initial SP axis. Initial SP for SVR and DeepNet will be same for each dataset as mentioned in.

### 5.1. Multi-dimensional bagging (Mdb): Implementation setup

This section describes the implementation details of Tier-1 of proposed framework. For this purpose, m-dimensional grid is formed for each base algorithm. From, it is cleared that, 3-dimensional, 4-dimensional and 2-dimensional grids are formed for RF, SVR and DeepNet respectively, which will serve as their initial SP.

**Fig 4:**
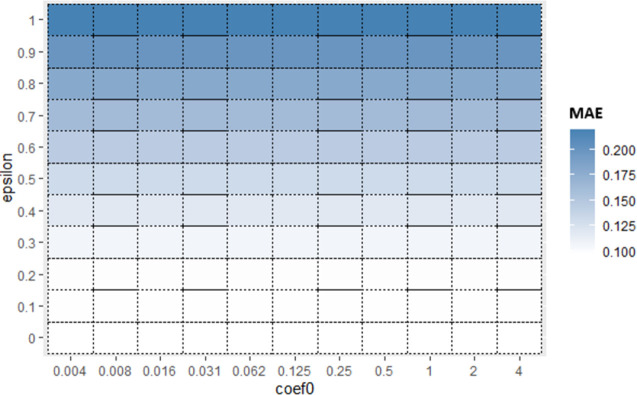
Initial SP of SVR (using *epsilon* and *coef*0).

**Fig 5:**
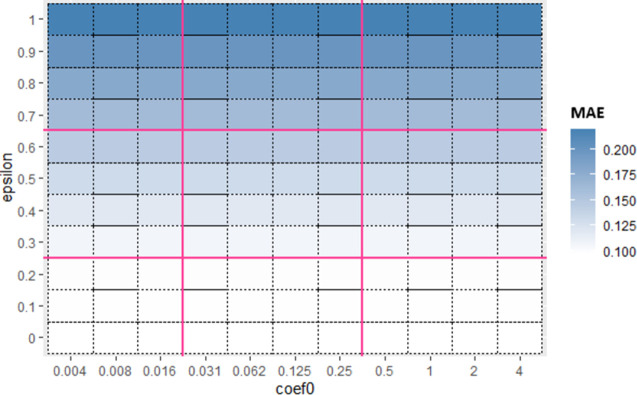
Sub_SP of SVR (using *epsilon* and *coef*).

Now for each base algorithm, Sub_SPs are created by dividing their initial SP as mentioned in Algorithm 1. Due to limitation of space, working of first 9 Sub_SPs of SVR, on Albrecht dataset is explained further in this section. Fig 4 represents one view of initial SP of SVR using *epsilon* and *coef*0 hyperparameters. From this initial SP, Sub-SPs1 to 9 are formed, represented in Fig 5. Red line shown in Fig 5 illustrates division of initial SP according to Algorithm 1. These Sub_SPs are used to create bags in proposed Mdb. Since performance of bagging algorithm is largely affected by appropriate number of bags. Hence for achieving sufficient number of bags, Sub_SPs needed to be increased. For this purpose, 2 hyperparameters are considered at a time.

Hence, in Sub-SPs 1 to 9, ranges for *epsilon* and *coef*0 are taken from division mentioned in Fig 5 and ranges of remaining hyperparameters *cost* and *gamma* are kept same as initial SP (i.e., *cost* [1,100] and *gamma* [0.001,1]). For Sub_SP 1 to 9, Table 5 represents Sub_SP range for each hyperparameter. These Sub_SP ranges serve as lower and upper bound for implementing FFA. FFA gives optimized value for each hyperparameter coming from their Sub_SP range (shown in Table 5 under column “Optimized”).

**Table 5:** Sub_SPs ranges and optimized values of SVR hyperparameters.

	*coef*0		*epsilon*		*gamma*		*cost*	
	Sub_SP range	Optimized	Sub_SP range	Optimized	Sub_SP range	Optimized	Sub_SP range	Optimized
Sub_SP1	[0.004, 0.016]	0.0804	[0.1, 0.2]	0.8	[0.001, 1]	0.004	[1, 100]	5
Sub_SP2	[0.004, 0.016]	0.0590	[0.3, 0.6]	0.8	[0.001, 1]	0.008	[1, 100]	9
Sub_SP3	[0.004, 0.016]	1.9894	[0.7, 1]	0.7	[0.001, 1]	0.074	[1, 100]	6
Sub_SP4	[0.031, 0.25]	0.2423	[0.1, 0.2]	0.6	[0.001, 1]	0.003	[1, 100]	49
Sub_SP5	[0.031, 0.25]	0.1370	[0.3, 0.6]	0.5	[0.001, 1]	0.009	[1, 100]	20
Sub_SP6	[0.031, 0.25]	3.5713	[0.7, 1]	0.6	[0.001, 1]	0.093	[1, 100]	20
Sub_SP7	[0.5, 4]	0.6643	[0.1, 0.2]	0.6	[0.001, 1]	0.003	[1, 100]	93
Sub_SP8	[0.5, 4]	0.0731	[0.3, 0.6]	0.5	[0.001, 1]	0.006	[1, 100]	83
Sub_SP9	[0.5, 4]	3.7803	[0.7, 1]	0.5	[0.001, 1]	0.064	[1, 100]	63

**Fig 6:**
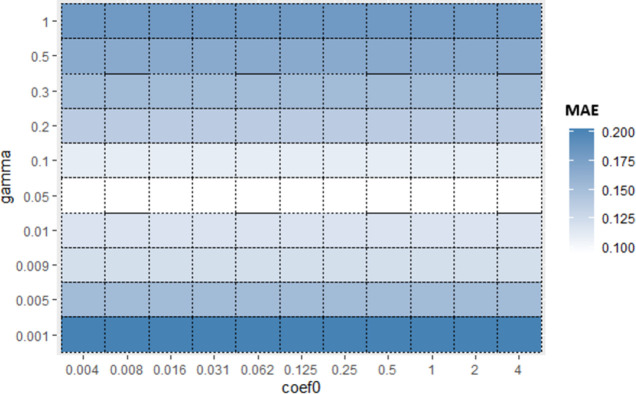
Initial SP of SVR (using *gamma* and *coef*0).

**Fig 7:**
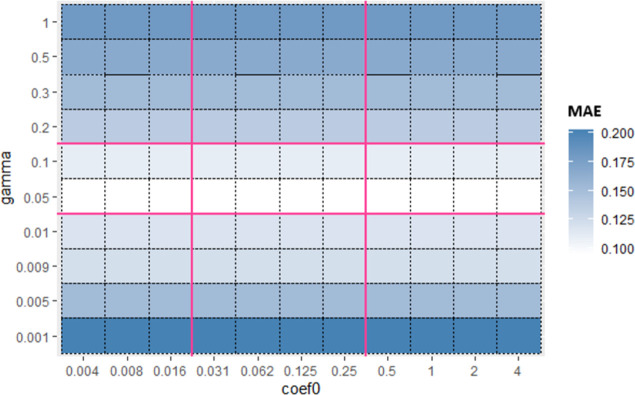
Sub_SP of SVR (using *gamma* and *coef*0).

**Fig 8:**
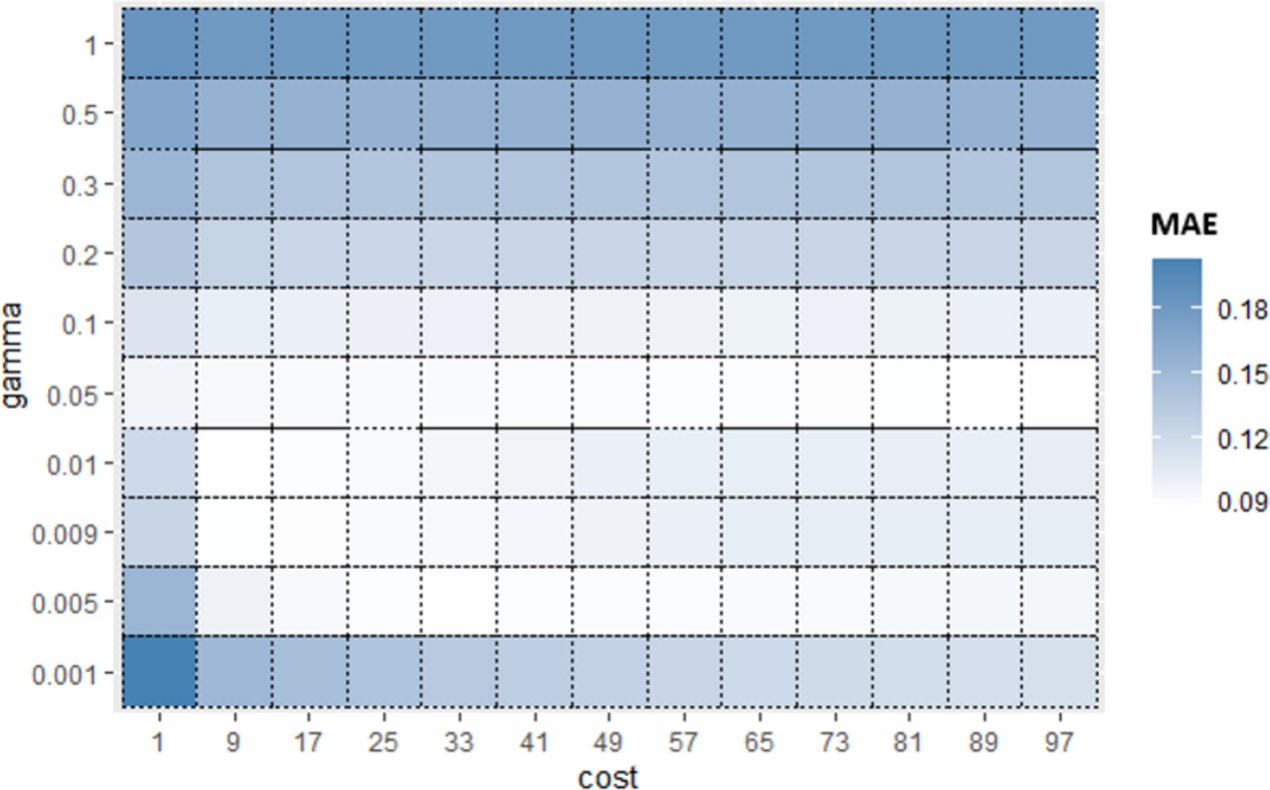
Initial SP of SVR (using *gamma* and *cost*).

**Fig 9:**
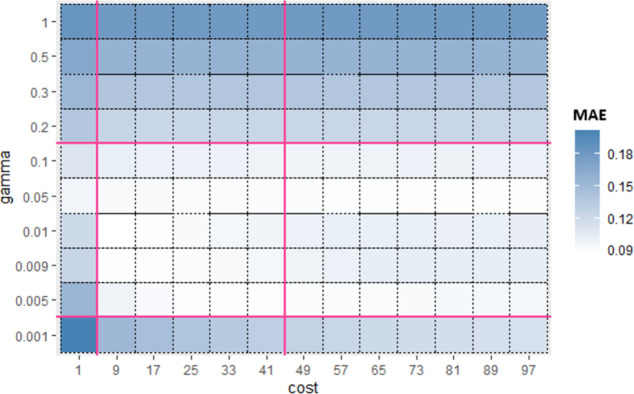
Sub_SP of SVR(using *gamma* and *cost*).

**Fig 10:**
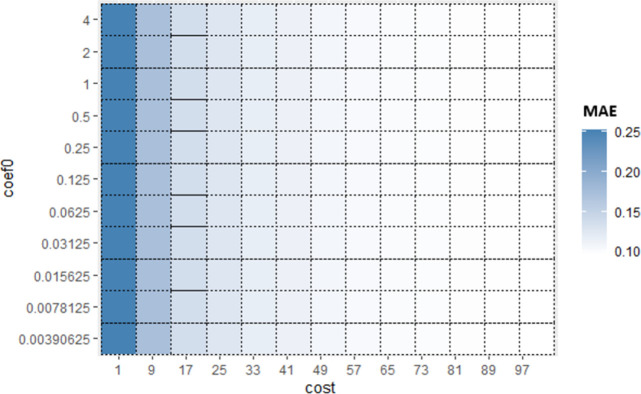
Initial SP of SVR (using *coef*0 and *cost*).

**Fig 11:**
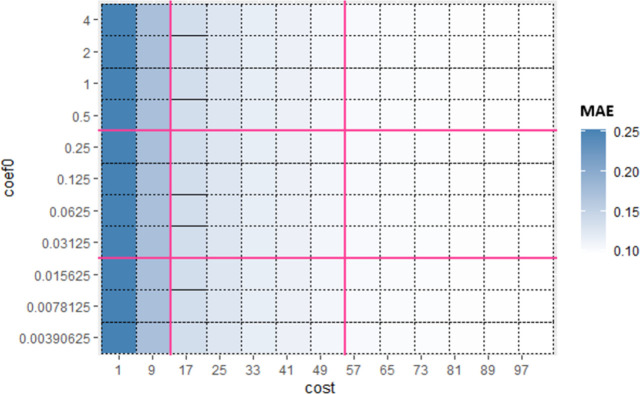
Sub_SPs of SVR (using *coef*0 and *co*.

**Fig 12:**
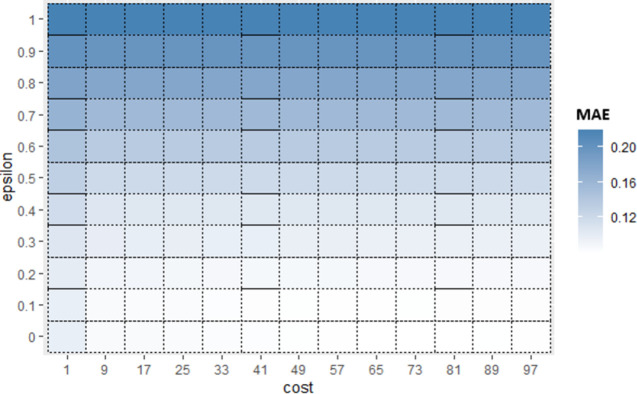
Mdb and MoWE performance on Albrecht dataset.

**Fig 13:**
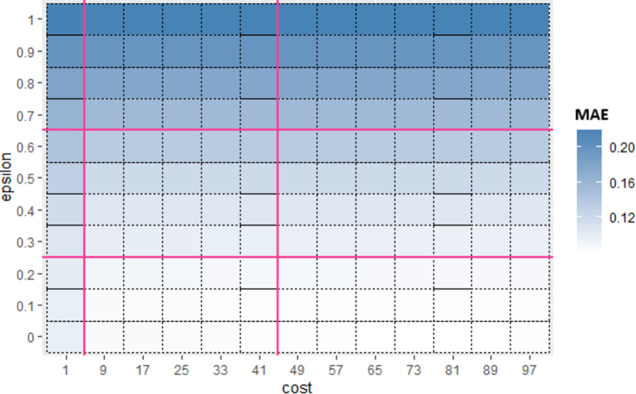
Mdb and MoWE performance on Albrecht dataset.

**Fig 14:**
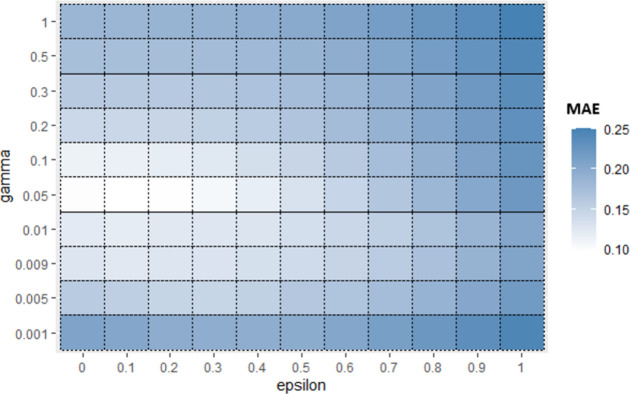
Mdb and MoWE performance on Albrecht dataset.

**Fig 15:**
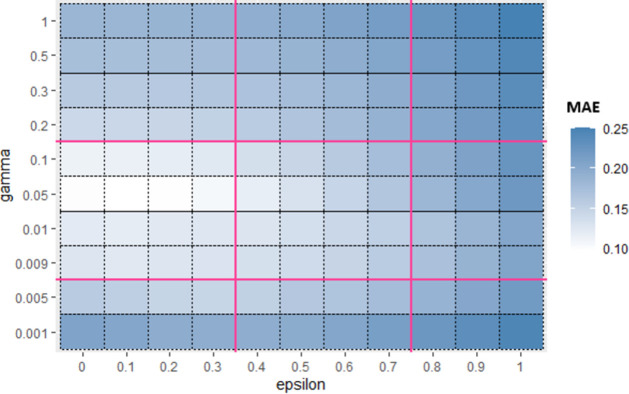
Mdb and MoWE performance on Albrecht dataset.

Similarly, for Sub_SPs 10 to 18, combination of other two hyperparameters (*gamma* and *coef*0) is taken (Fig 6) and their Sub_SPs are formed (Fig 7). For Sub_SPs 10 to 18, remaining two hyperparameters (*epsilon* and *cost*) have ranges same as their initial SPs (Table 6). Same process is repeated for all combinations of SVR hyperparameters. Figs 8 and 9 represent Initial SPs and Sub_SP respectively for hyperparameters (*gamma*, *cost*). Similarly, Figs 10 and 11 show Initial SP and Sub_SP for hyperparameters (*coef*0, *cost*). Figs 12 and 13 for hyperparameters (*epsilon*,*cost*), and Figs 14 and 15 for hyperparameters (*gamma*,*epsilon*), respectively.

**Table 6:** Initial SPs of three base algorithm.

Techniques	Parameters	Initial SP	
		Lower Bound	Upper Bound
RF	*mtry*	1	F-1; F = No. of Features
	*ntree*	1	500
	*nodesize*	1	Total No. of samples
SVR	*gamma*	0.001	1
	*coef*0	0.0039	4
	*cost*	1	100
	*epsilon*	0.1	0.9
DeepNet	*size*	1	10
	*decay*	0.001	0.5

After applying above mentioned process for all possible combinations of SVR hyperparameters, 54 Sub_SPs are formed (in this paper, we mentioned only first 9 Sub_SPs due to limitation of space). These Sub_SPs are fed into FFA for getting best hyperparameters residing in each Sub_SP. Hence, total of 54 Sub_SPs are fed into FFA individually, to get 54 sets of optimized hyperparameters of SVR. These 54 sets of FFA-optimized hyperparameters are used to train 54 bags of SVR, which are then combined to get finalized Mdb scheme for SVR named as *SVR_Mdb*. Mdb schemes for RF and DeepNet are obtained using the same process and referred as RF_Mdb and DeepNet_Mdb respectively. Descriptive statistics of optimized hyperparameters for three solo learners (RF, SVR and DeepNet) are listed in Appendix A: Table 12 in S1 File.

### 5.2. Dataset

Implementation of proposed effort estimation scheme is performed on 8 datasets, available on SEACRAFT [63] repository. Software Engineering Artifacts Can Really Assist Future Tasks (SEACRAFT) is publicly available online data repository (formerly known as PROMISE [64]. Selected dataset for this study are: Albrecht [65] Deharnais [66] Miyazaki [67], China [68], Cocomo81 [69], Finnish [70], Kitchenham [71] and Maxwell [72].

These datasets were selected due to their frequent utilization in evaluating ensemble effort estimation techniques [6]. Table 7 summarizes descriptive statistics of all selected datasets including, number of projects each dataset, number of features, effort measurement unit along with median, mean, minimum, maximum, skewness and kurtosis measures of effort. As it is clear from Table 7, effort values of all datasets do not follow a normal distribution since skewness values ranged from 2.03 to 10.64 [73]. This is rendered using min-max normalization rule to keep degree of influence similar for all models. Reason for applying normalization first is that, although selected ML techniques work with any data distribution, but effort prediction is more accurate when values of output variable (effort) are symmetrically concentrated around the mean [74].

**Table 7:** Descriptive and statistical details of effort estimation datasets used.

Dataset	#Proj.	Effort Unit	#Features	Effort Statistics					
				Min	Max	Mean	Median	Skewness	Kurtosis
Albrecht	24	MM	7	0.5	105	21.87	11 2	2.30	4.7
Desharnais	77	MH	11	546	23940	4833.90	3542	2.03	5.3
Miyazaki	48	MM	8	5.6	1586	87.47	38	6.26	41.3
China	499	MH	18	26	54620	3921.04	1829	3.92	19.3
Cocomo81	63	MM	17	5.9	11400	683.3	98	4.26	19.35
Finnish	38	MH	8	6.13	10.19	8.39	8.59	-0.29	-1.19
Kitchenham	145	MH	4	219	113930	3113.11	1557	10.64	119.59
Maxwell	62	MH	26	583	63694	8223.21	5189.5	3.18	12.02

### 5.3. Comparison models

We compared our proposed MoMdbWE (Mdb with MoWE) technique with multiple effort estimation methods. First, proposed Mdb schemes of all three models are compared with their respective solo base algorithms (RF, SVR, DeepNet), to verify improvement of proposed *Multi-dimensional bagging* over individual learners. Also, three Mdb schemes are compared with bagging ensemble [8] to confirm if Mdb provide better prediction than normal bagging.

After that, proposed MoWE model is compared with other heterogenous ensemble methods found in literature, including gradient boosting [75] stacking [76] majority voting and weighted ensemble (with non-optimized weights) [30]. Further, performance comparison of our proposed MoMdbWE techniques is made with previous EEE studies (Table 11).

### 5.4. Performance evaluation measures

This section includes performance criteria used in this study to evaluate proposed MoMdbWE technique in comparison with the models (mentioned in Section 5.3: Comparison models). Performance measures (error metrices) used in the study are: Mean absolute error (MAE), Root mean square error (RMSE), Magnitude of relative error (MRE), Mean magnitude of relative error (MMRE), Median of magnitude of relative error (MdMRE) and Pred(l), which are widely used evaluation metrices in SDEE literature [77]. SA and Δ are another important evaluation measures to analyze performance of effort estimation model. Many SDEE studies declared MMRE as unreliable measure for prediction [25, 78] due to its biasness towards underestimation and asymmetric nature. For this reason, SA and Δ are also incorporated in this study, proposed by Shepperd and McDonell [25]. Details of evaluation measures are as follows:

1. MAE is calculated by taking average absolute difference between estimated effort and the effort observed, represented by Eq 17
$$\text{MAE}=\frac{1}{N}\sum_{n=1}^{N}{|\text{Actual}\;\text{Effort}}_{\text{n}}-{\text{Estimated}\;\text{Effort}}_{\text{n}}|$$ (Eq 17)
2. RMSE is defined as the standard deviation of the differences between actual and calculated effort given by Eq 18
$$\text{RMSE}=\sqrt{\frac{\sum_{n=1}^{N}{\left({\text{Actual}\;\text{Effort}}_{\text{n}}-{\text{Estimated}\;\text{Effort}}_{\text{n}}\right)}^{2}}{N}}$$ (Eq 18)
3. The MRE for finding the relative error between actual effort values and observed effort values for each project of dataset is calculated as given in Eq 19.
$$\text{MRE}=\frac{|\text{Actual}\;\text{Effort}-\text{Estimated}\;\text{Effort}|}{\text{Actual}\;\text{Effort}}$$ (Eq 19)
4. MMRE is calculated by taking average of MRE’s of all data samples, as given in Eq 20.
$$\text{MMRE}=\frac{1}{N}\sum_{n=1}^{N}{\text{MRE}}_{\text{n}}$$ (Eq 20)
5. MdMRE is the median of MREs of all projects of a dataset as shown in Eq 21
$$\text{MdMRE}=\text{median}\left({\text{MRE}}_{\text{n}}\right)$$ (Eq 21)
  Pred(l) is the count of MRE less than or equal to a value (l), for all data points, represented as Eq 22
$$Pred(l)=\frac{1}{N}\overset{N}{\underset{n=1}{\sum }}\left\{\begin{array}{c} 1\;\mathrm{if}\;\text{MRE}\leq \text{l}\\ 0\;\text{otherwise} \end{array}\right.$$ (Eq 22)
  Where N is total number of project instances, l is taken as 0.25, so PRED (0.25) will be calculated as total number of projects having MRE less than or equal to 0.25. An effort estimation model is considered satisfactory in terms of accuracy if MRE is less than 0.25 and PRED (25) is greater than 0.75 [3].
6. Standard accuracy (SA) reflects the percentage by which an effort estimation gives accurate prediction, in comparison with random guessing. SA is calculated based on MAE of a model. Effect size (Δ) is used to verify whether estimate of a models, occurred by chance and is there any improvement observed over random guessing. SA and Δ are shown in Eqs 23 and 24 respectively.
$$SA=1-\left(\frac{MAE\rho_{t}}{MAE\rho_{0}}\right)$$ (Eq 23)
$$\Delta =\left(\frac{MAE\rho_{t}-MAE\rho_{0}}{S\rho_{0}}\right)$$ (Eq 24)
  Where $MAE\rho_{t}$ is *MAE* of the estimation method *ρ*_*t*_, and $MAE\rho_{0}$ is mean of 1000 random guessing samples. $MAE\rho_{0}$ is defined as “predicted effort of a target task by randomly choosing training instance with equal probability over all remaining *T* − 1 samples”. Assign Estimated Effort_t_ = Estimated Effort_r_ where *r* is randomly chosen from $1\ldots T\wedge \text{r}\neq \text{t}.S\rho_{0}$ is standard deviation of random guessing samples. Further, Shepperd and McDonell [25] recommended using 5% quantile of random guessing while estimating a technique, to ensure likelihood of non-random prediction. SA near to zero reflects poor performance of model than random guessing, SA near to 1 ensures better performance while, negative SA values are worrisome [25]. Absolute value of Δ are interpreted in terms of small (≃ 0.2), medium (≃ 0.5) and large (≃ 0.8) categories as proposed by Cohen [79]. Values of Δ > 0.5 reflects better performance of a model.

### 5.5. Statistical evaluation

Statistical evaluation is very crucial part of any research, since it provides evidence that performance achieved by a model is significant enough to be compared. To ensure if proposed model’s results are statistically significant than other comparing models [80], Wilcoxon test is used [81]. Wilcoxon test is included to verify the significance difference between error measures of all comparing models. The p-value (significance level) of Wilcoxon test evaluates that the results of a model are not accumulated by chance. Wilcoxon test belongs to non-parametric statistical test category, i.e., it makes no assumptions about the probability distributions of the variables under test.

In this study, Wilcoxon test is implemented with Bonferroni correction [82] and significance level (α = 0.05) is applied to minimize Type I error [83]. For each dataset, statistical test is performed on absolute errors accumulated from testing data samples. For each pairwise comparison, samples of absolute error are taken from same test set.

The null and alternative hypothesis of significance test are shown in Eq 25:

$$\left\{\begin{array}{l} H_{0}:\mu_{k}=\mu_{m}\;OR\;\mu_{k}-\mu_{m}=0\\ H_{1}:\mu_{k}\neq \mu_{m}\;OR\;\mu_{k}-\mu_{m}\neq 0 \end{array}\right.$$ (Eq 25)

Null hypothesis (H_o_): Difference between error means of two compared models is zero, i.e., a model_k_ (with error mean μ_k_) have similar performance compared to model_m_ (with error mean μ_m_)

Alternative hypothesis (H_1_): Difference between error means of two compared models is not zero i.e., a model_k_ doesn’t hold equal performance model_m_.

## 6. Results

This section presents results of the experiments performed in this study to compare performance and result accuracy of proposed Mdb scheme and MoWE to other models.

Firstly, the performance of FFA (in terms of hyperparameter optimization) is evaluated in comparison with GS method and two metaheuristic algorithms GA and PSO. Hyperparameters of RF, SVR and DeepNet are optimized with GS, GA, PSO and FFA using their initial SPs (Table 5). Results are shown in Appendix A: Table 12 in S1 File, with MAE and elapsed time (in seconds) taken by each optimization algorithm. Results show that FFA provides good fitness in all three base algorithms. Also, GS proves time consuming, in all datasets, so GS is not good option in executing large set of experiment as proposed framework entails. GA, PSO show comparatively less execution time for SVR and DeepNet in some cases, but performance of FFA is still above for all base algorithms.

After verifying performance of FFA (compared to other optimization algorithms), proposed Mdb and MoWE scheme is implemented using FFA hyperparameter and ensemble weight optimization. For Mdb scheme, descriptive statistics of RF, SVR and DeepNet hyperparameters coming from each Sub-SP division, are given in Appendix A: Table 12 in S1 File.

### 6.1. Results of proposed Multi-dimensional bagging (Mdb) and Metaheuristic-optimized weighted ensemble (MoWE)

Evaluation results of proposed *Multi-dimensional bagging (Mdb)* and *Metaheuristic-optimized weighted ensemble (MoWE)* are discussed in this section. listed SA and effect size (Δ) measures of all compared models.

Firstly, we will discuss the performance of all three Mdb schemes. As shown in the,. *Mdb schemes* achieved compelling estimation accuracy in all datasets. In terms of SA, RF_Mdb, SVR_Mdb and DeepNet_Mdb outperformed their solo learners (RF, SVR and DeepNet) as well as their normal bagging versions. If we look closely to SA values of, there are some case where solo learners surpassed their bagging versions. For instances, RF surpassed RF_Bagging for Albrecht, China and Finnish datasets, and both algorithms gave equal performance in Desharnais, Miyazaki, Kitchenham and Maxwell datasets. Similarly, SVR gave better performance than SVR_Bagging in Albrecht, China, COCOMO81 and Finnish datasets, while both are equal in Miyazaki dataset. DeepNet, however surpassed from DeepNet_Bagging in Albrecht, Miyazaki, Finnish and Kitchenham datasets while no equal performance in observed for both. Again, it is worth mentioning, no solo algorithm or bagging algorithm surpassed or gave equal performance in comparison with Mdb schemes i.e., Mdb schemes of all three solo learners (RF_Mdb, SVR_Mdb and DeepNet_Mdb) gave significantly improved performance compared to solo and bagging algorithms.

Results of also confirm that, performance of effort prediction further improvised when individual Mdb schemes are combined using FFA-optimized weights to form MoWE. SA of proposed MoWE is giving good performance evaluation in comparison with solo algorithms as well as other heterogenous ensembles. Moreover, MoWE in all datasets performed better then all three Mdb schemes. Among heterogenous ensembles (apart from MoWE), Majority voting has shown second best considerable performance in terms of SA for Albrecht, Desharnais, Miyazaki, Cocomo81, Kitchenham datasets, while Stacking is second best technique in China, Finnish and Maxwell datasets.

As discussed in Section 5.4: Performance evaluation measures, performance of effect size is interpreted as small (≃ 0.2), medium (≃ 0.5) and large (≃ 0.8) categories. Corresponding to the SA performance, similar results are observed in terms of Δ measure, where absolute values of Δ of all Mdb schemes are higher than solo learners and normal bagging.

**Fig 16:**
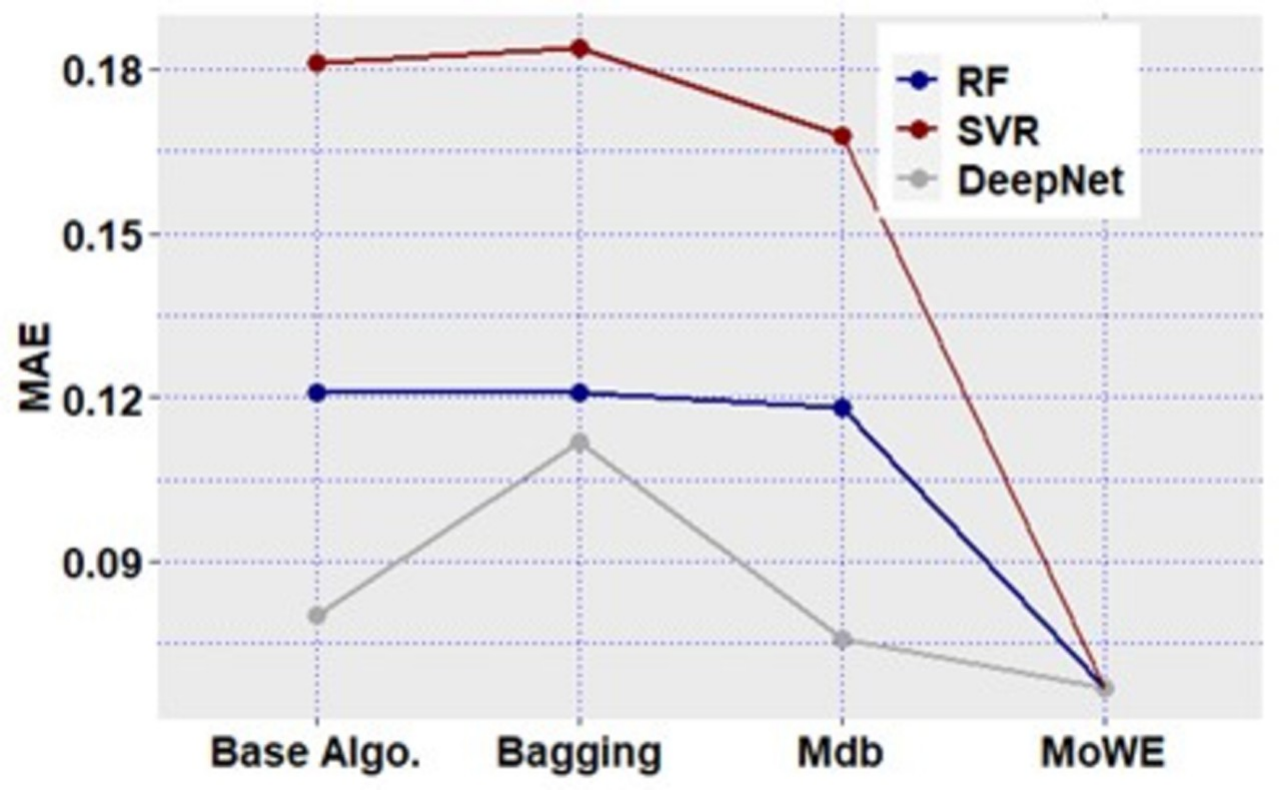
Mdb and MoWE performance on Albrecht dataset.

**Fig 17:**
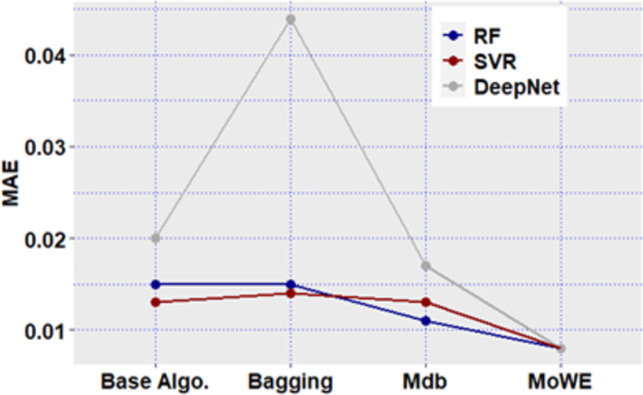
Mdb and MoWE performance on Albrecht dataset.

**Fig 18:**
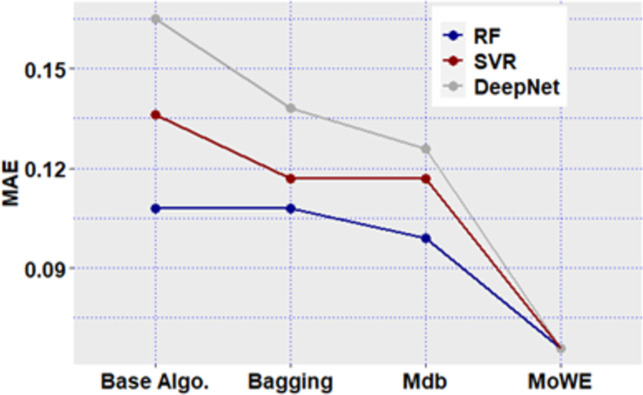
Mdb and MoWE performance on Albrecht dataset.

**Fig 19:**
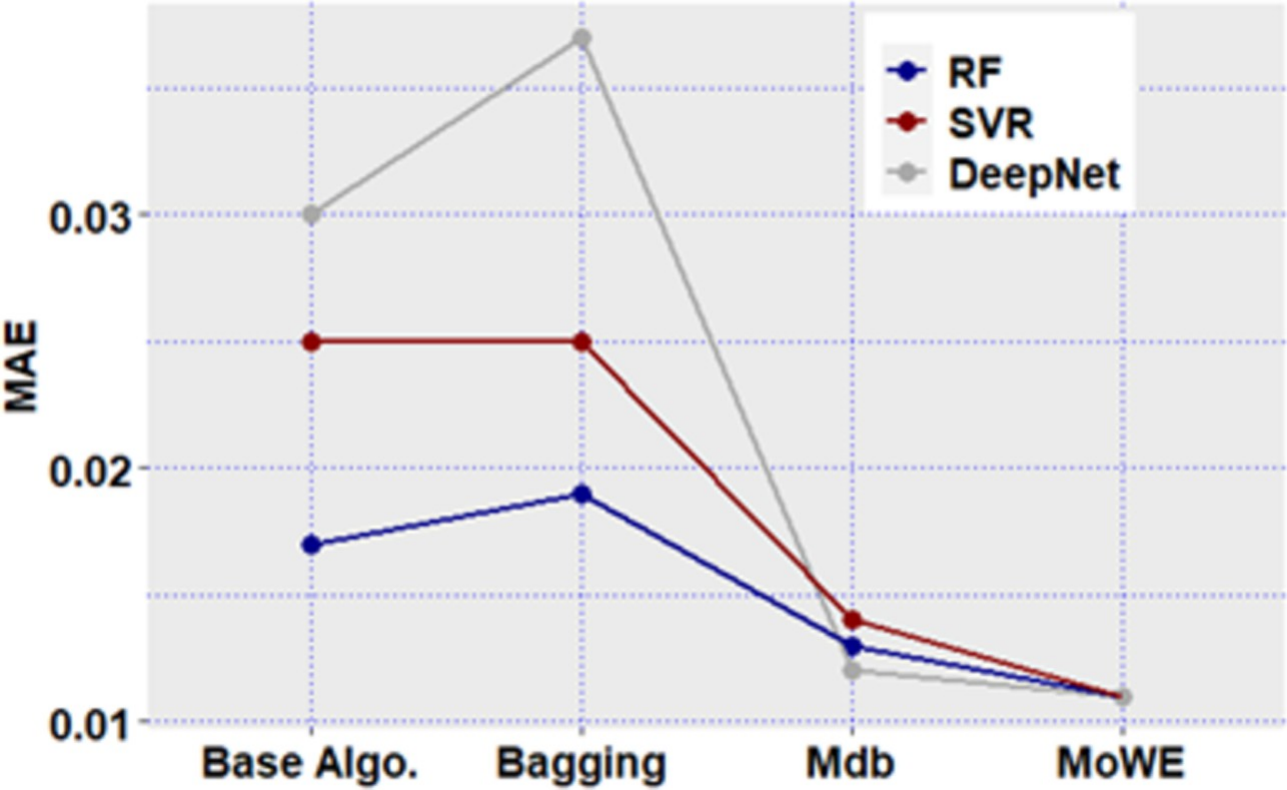
Mdb and MoWE performance on Albrecht dataset.

**Fig 20:**
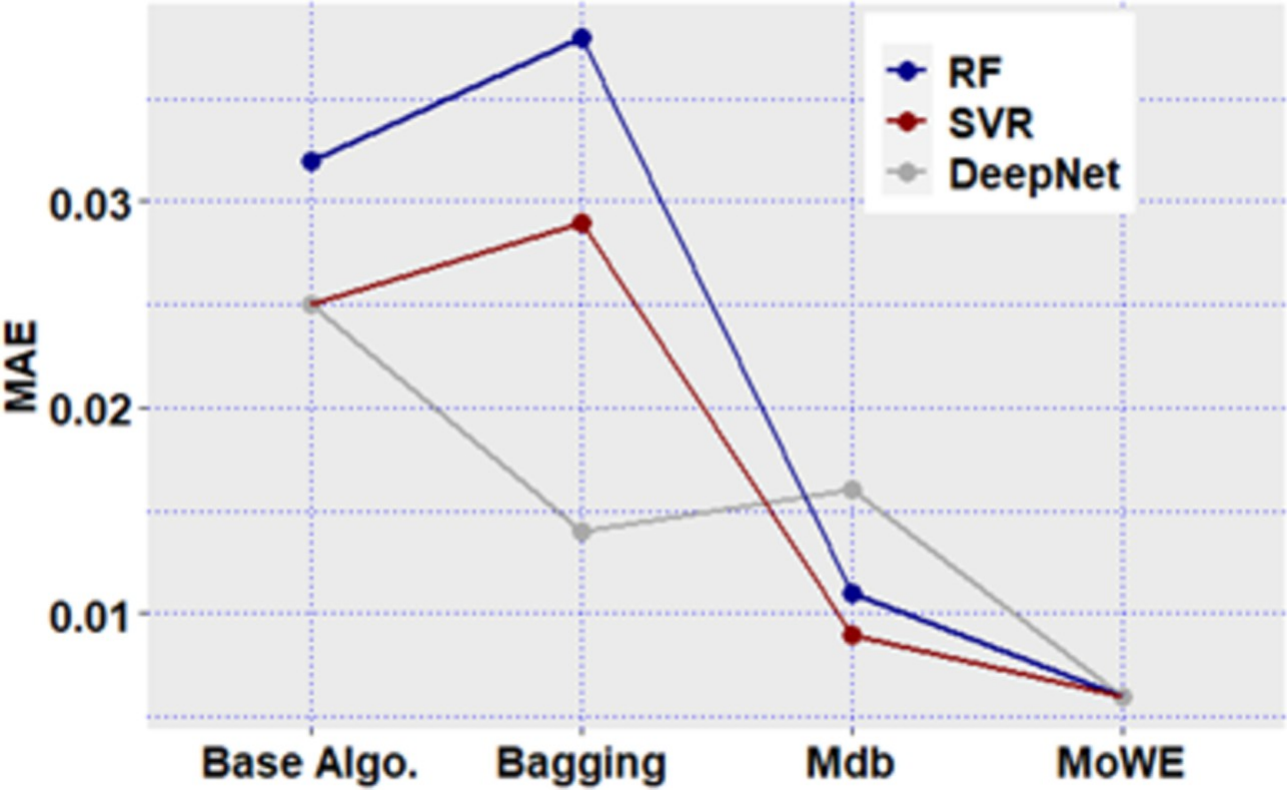
Mdb and MoWE performance on Cocomo81 dataset.

**Fig 21:**
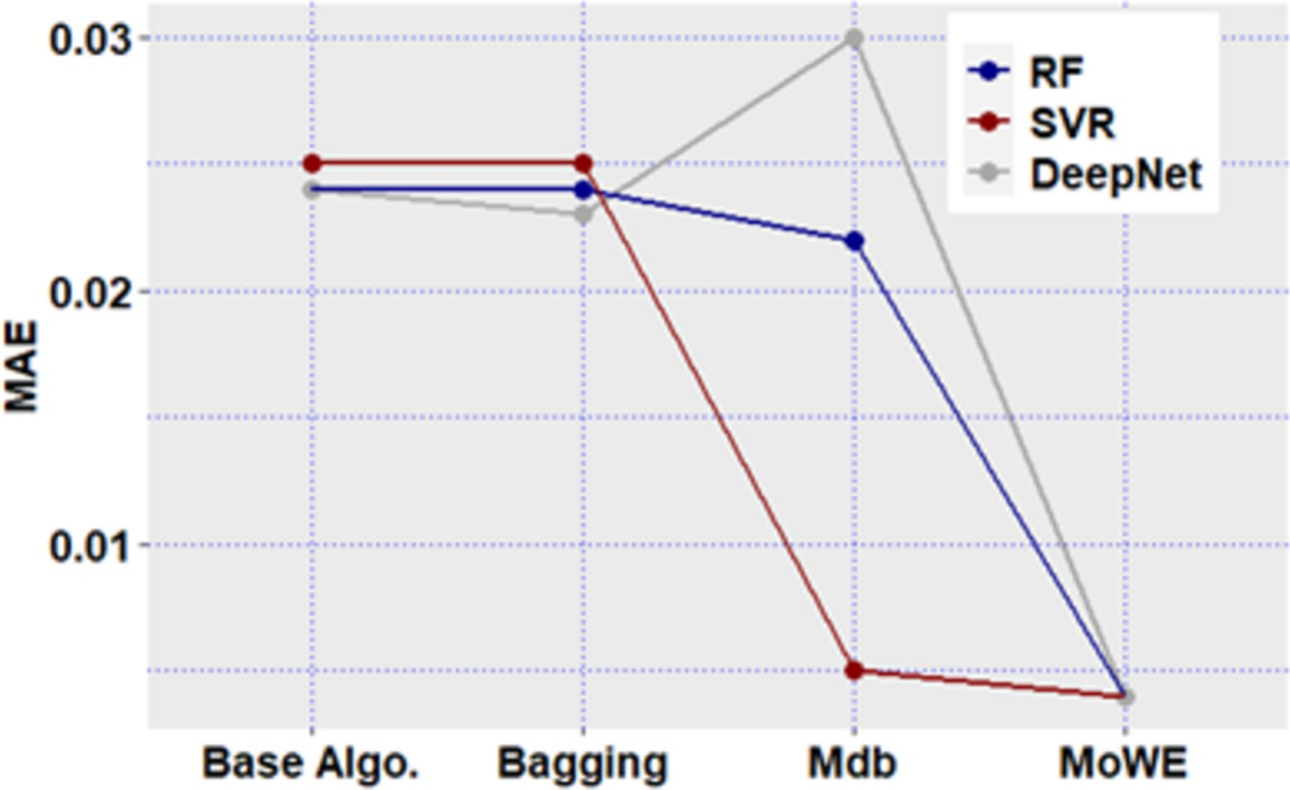
Mdb and MoWE performance on Finnish dataset.

**Fig 22:**
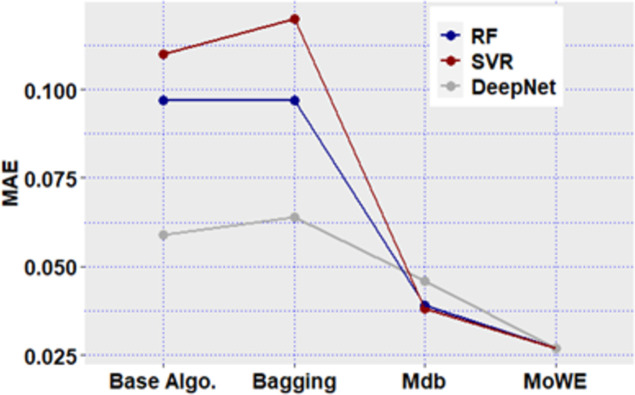
Mdb and MoWE performance on Kitchenham dataset.

**Fig 23:**
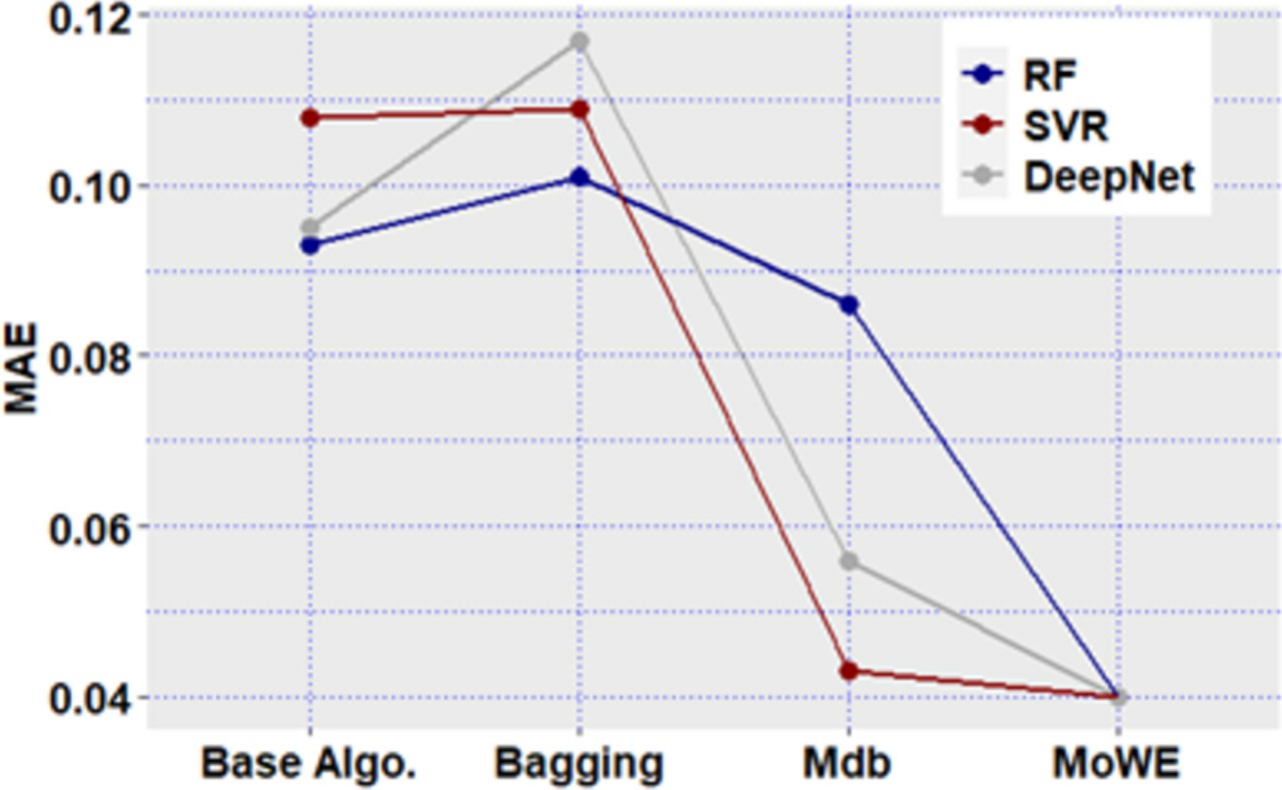
Mdb and MoWE performance on Maxwell dataset.

For Albrecht, however, models shown effect size between small to medium category, except SVR and SVR_Bagging, which shows performance even less than small effect size category (i.e., Δ < 0.2). No models trained on Albrecht fall under large effect size. For Desharnais datasets, Δ lie between small to medium category only, showing quite moderate performance. For Miyazaki datasets, Δ values observed for all models are of large effect size except DeepNet and DeepNet bagging, which still lie under moderate Δ range. China dataset, showing 10 out of 14 models with moderate effect size category, while in Cocomo81, half models are observing medium Δ range. For Finnish dataset, all models found are in large to medium category (except Majority voting ensemble). Model trained on Kitchenham and Maxwell show small to very small effect size category. Overall, for Miyazaki and China dataset, models with good effect size range are observed. Proposed MoWE ensemble has shown medium to larger Δ category in all datasets (expect Kitchenham). MAE performance of Mdb schemes and MoWE is also expressed graphically in Figs 16–23 for all datasets. Clear, there is a decreasing trend in error (MAE) from base algorithms to Mdb schemes, to MoWE.

### 6.2. Result of statistical evaluation

For statistical evaluation, Wilcoxon test is performed based on absolute errors of all compared models. For reporting statistical test results, post-hoc analysis with Bonferroni correction [82] is performed by pairwise comparison. For tier 1, comparison is made between:

- Three Mdb schemes (RF_Mdb, SVR_Mdb and DeepNet_Mdb) and their respective base algorithms.
- Mdb schemes with normal bagging

For tier 2, Wilcoxon test with post-hoc analysis performed between MoWE and:

- Three base algorithms
- Normal bagging schemes of three solo models (RF_bagging, SVR bagging, DeepNet bagging)
- Three Mdb schemes (Mdb_RF, Mdb_SVR, Mdb_DeepNet
- Other heterogenous ensemble models.

The reason for this comparison is to justify, whether performance of Mdb schemes is significantly improved compared to their base algorithms and normal bagging versions of base algorithm. p-value of Wicoxon comparison is reported in Since no base algorithm and bagging algorithm outperformed in terms of SA and effect size on any dataset (Table 8), pairwise comparison of RF, SVR, DeepNet and bagging versions of these algorithms is not added.

**Table 8:** SA and effect size evaluation of Proposed Mdb and MoWE.

	Albrecht		Desharnais		Miyazaki		China		Cocomo81		Finnish		Kitchenham		Maxwel	
	SA	Δ	SA	Δ	SA	Δ	SA	Δ	SA	Δ	SA	Δ	SA	Δ	SA	Δ
RF	0.48	-0.41	0.53	-0.46	0.92	-0.81	0.81	-0.63	0.68	-0.43	0.70	-0.78	0.43	-0.18	0.31	-0.27
RF_Bagging	0.41	-0.41	0.53	-0.46	0.92	-0.81	0.79	-0.62	0.71	-0.44	0.64	-0.71	0.43	-0.18	0.31	-0.27
RF_Mdb	0.49	-0.43	0.57	-0.50	0.95	-0.83	0.86	-0.67	0.88	-0.55	0.90	-1.00	0.48	-0.20	0.36	-0.31
SVR	0.22	-0.19	0.41	-0.36	0.94	-0.82	0.72	-0.56	0.77	-0.48	0.66	-0.73	0.39	-0.17	0.21	-0.18
SVR_Bagging	0.21	-0.18	0.50	-0.43	0.94	-0.81	0.71	-0.55	0.73	-0.46	0.53	-0.59	0.40	-0.17	0.22	-0.19
SVR_Mdb	0.28	-0.24	0.50	-0.43	0.95	-0.82	0.85	-0.66	0.95	-0.59	0.89	-0.99	0.70	-0.30	0.80	-0.69
DeepNet	0.66	-0.57	0.29	-0.25	0.91	-0.79	0.51	-0.40	0.44	-0.28	0.82	-0.91	0.28	-0.27	0.04	-0.03
DeepNet_Bagging	0.52	-0.45	0.41	-0.35	0.81	-0.70	0.61	-0.47	0.87	-0.54	0.76	-0.85	0.51	-0.22	0.15	-0.13
DeepNet_Mdb	0.67	-0.58	0.46	-0.40	0.93	-0.80	0.87	-0.68	0.88	-0.55	0.86	-0.96	0.63	-0.12	0.56	-0.48
Gradient Boosting	0.44	-0.38	0.29	-0.25	0.94	-0.82	0.74	-0.58	0.52	-0.32	0.02	-0.13	0.41	-0.18	0.28	-0.24
Stacking	0.41	-0.35	0.41	-0.35	0.94	-0.81	0.81	-0.63	0.62	-0.39	0.82	-0.92	0.50	-0.21	0.48	-0.41
Majority Voting	0.49	-0.43	0.46	-0.40	0.95	-0.83	0.53	-0.41	0.87	-0.55	0.26	-0.29	0.53	-0.23	0.05	-0.04
Weighted ensemble	0.42	-0.37	0.37	-0.32	0.56	-0.48	0.28	-0.22	0.85	-0.53	-0.21	0.24	0.27	-0.11	0.03	-0.20
Proposed MoWE	0.71	-0.62	0.72	-0.62	0.97	-0.84	0.89	-0.69	0.93	-0.58	0.88	-0.98	0.83	-0.35	0.88	-0.76

Results of Wilcoxon comparison for Tier-2 is reported in. As discussed earlier, Wilcoxon test is performed on significant level α = 0.05 for statistically evaluating all model. However, models showing significance at α = 0.1 are denoted by (*). Analyzing the statistical results of Tier-1(Table 9) it is clear that RF_Mdb is showing significance at 0.05, compared to RF and RF_Bagging in all datasets, expect for Desharnais, where RF_Mdb and solo RF are significantly different but only at α = 0.1.

**Table 9:** Wilcoxon test results comparing Mdb schemes with solo learners and normal bagging algorithm.

Techniques	Wilcoxon test p-value							
	Albrecht	Desharnais	Miyazaki	China	Cocomo81	Finnish	Kitchenham	Maxwell
RF_Mdb Vs RF	6.22e-04	0.092 *	0.029	3.10e-08	1.27e-03	0.017	5.14e-03	4.02e-03
RF_Mdb Vs RF_Bagging	6.22e-04	3.11e-04	0.019	5.56e-10	4.79e-04	6.81e-03	3.70e-03	0.037
SVR_Mdb Vs SVR	0.021	1.97e-03	7.12e-05	2.03e-04	1.00e-03	0.021	1.43e-04	0.011
SVR_Mdb Vs SVR_Bagging	0.065 *	1.10e-03	1.40e-04	1.23e-03	5.43e-04	0.017	2.71e-04	8.25e-03
DeepNet_Mdb Vs DeepNet	1.55e-04	0.038	1.29e-08	1.19e-13	4.18e-03	2.958e-06	1.02e-08	0.043
DeepNet_Mdb Vs DeepNet Bagging	0.1049 *	2.16e-03	1.29e-08	3.94e-15	0.019	3.328e-05	6.28e-09	0.014

*Significance at 0.1

Similarly, in Albrecht dataset, SVR_Mdb and DeepNet_Mdb models are showing significance over normal bagging at α = 0.1. In all other models, Mdb schemes statistically outperformed (at α = 0.05) compared to their solo learners and normal bagging, hence rejecting the null hypothesis (Eq 25).

In statistical analysis of Tier-2 (Table 10), it is important to note that MoWE performed significantly well (at α = 0.05) compared to all solo leaners and normal bagging algorithms for all datasets. However, for Mdb schemes in some datasets, MoWE is showing lower significance. Particularly, comparing MoWE and SVR_Mdb, lower statistical significance is observed (i.e., at α = 0.1) for Cocomo81, Finnish, Kitchenham and Maxwell. It is worth mentioning that, MoWE showing difference at α = 0.05 compared to all models in Albrecht, Desharnais and Miyazaki datasets. In comparison with other heterogenous ensembles, significant improvement of MoWE is observed for all models in all datasets. For Cocomo81, Majority voting and Weighed ensemble are showing significance on only on α = 0.1. To conclude statistical significance exits in performance for proposed Mdb and MoWE technique either at α = 0.05 or α = 0.1. Hence performance superiority of proposed scheme is visible in all datasets, This rejects the null hypothesis stating that there is no difference in performance of proposed technique and other comparing models. Our experimental results clearly show that there exists statistical difference between proposed technique and other models either on significance level α = 0.05 or 0.1.

**Table 10:** Wilcoxon test results comparing MoWE with other models.

Techniques	Wilcoxon test p value							
	Albrecht	Desharnais	Miyazaki	China	Cocomo81	Finnish	Kitchenham	Maxwell
MoWE Vs RF	0.049	3.72e-12	3.16e-03	0.036	6.32e-06	0.017	0.011	0.039
MoWE Vs RF_Bagging	1.09e-03	0.029	4.27e-03	0.026	3.058e-05	1.03e-04	4.61e-03	7.47e-03
MoWE Vs RF_Mdb	0.045	0.03146	4.27e-03	0.084 *	0.102 *	0.024	3.91e-03	0.019
MoWE Vs SVR	0.038	0.038	0.021	0.031	6.83e-03	0.039	0.009	0.011
MoWE Vs SVR_Bagging	3.11e-04	9.26e-03	0.037	0.051	1.78e-03	0.033	0.034	6.20e-03
MoWE Vs SVR_Mdb	0.010	0.041	2.70e-03	0.012	0.0563 *	0.092 *	0.067 *	0.068 *
MoWE Vs DeepNet	1.09e-03	0.021	3.67e-03	3.98e-16	0.039	0.048	2.80e-03	0.013
MoWE Vs DeepNet_Bagging	6.22e-04	0.016	9.88e-03	< 2.2e-16	0.037	0.018	5.51e-04	2.48e-03
MoWE Vs DeepNet_Mdb	0.015	0.031	0.029	0.016	0.081 *	0.029	0.059 *	0.0354
MoWE Vs Gradient Boosting	0.011	4.81e-03	1.50e-04	2.2e-16	9.47e-11	3.37e-06	< 2.2e-16	1.63e-07
MoWE Vs Stacking	0.038	0.018	2.15e-04	0.038	1.99e-03	2.95e-06	0.047	0.029
MoWE Vs Majority Voting	0.021	0.012	5.78e-04	0.029	0.079 *	2.01e-04	2.62e-04	5.10e-03
MoWE Vs Weighted ensemble	6.99e-03	9.95e-04	9.88e-03	< 2.2e-16	0.070 *	2.74e-04	3.367e-06	2.16e-04

*Significance at α = 0.1

A comparative performance is also performed in this study, in which proposed model is compared with previous SDEE studies employing state-of-the-art ensemble learning algorithms, shown in. Table contains ensemble techniques, performance metrices, working dataset and performance achieved by the study under-consideration. Also, performance improvement achieved by our proposed MoMdbWE is listed in Table 11. It is clear from the table that proposed scheme showed improved results compared to other ensemble techniques in compared performance metrices.

**Table 11:** Comparative evaluation of past EEE work with proposed MoMdbWE scheme.

Ensemble Technique	Previous studies	Performance Metrices	Dataset	Performance	MoMdbWE improvement
RF	[84]	MMRE	Albrecht	0.73	68.2%
			Desharnais	0.42	27.1%
		MdMRE	Albrecht	0.60	71.3%
			Desharnais	0.32	7.5%
		Pred(0.25)	Albrecht	42.86	67.4%
			Desharnais	43.48	72.7%
Bagging	[85]	MMRE	Albrecht	0.60	61.3%
			Desharnais	0.50	38.8%
			Miyazaki	0.56	23.2%
	[10]	Pred(0.25)	Miyazaki	0.20	335.0%
Boosting	[85]	MMRE	Albrecht	0.83	72.0%
			Desharnais	0.41	25.4%
			Miyazaki	0.83	48.2%
Stacking	[35]	MMRE	Desharnais	0.42	27.1%
			Miyazaki	0.54	20.4%
		MdMRE	Desharnais	0.38	22.1%
			Miyazaki	0.43	9.3%
		Pred(0.25)	Desharnais	0.44	72.7%
			Miyazaki	0.47	85.1%
Majority Voting	[10]	MMRE	Albrecht	0.67	65.4%
			Desharnais	0.509	39.9%
			Miyazaki	0.66	34.8%
	[27]	Pred(0.25)	Albrecht	37.50 45.83	89.5% 56.5%
Weighted Ensemble	[35]	MMRE	Desharnais	0.26	74.6%
			Miyazaki	0.50	80.8%
	[27]	MdMRE	Desharnais	0.44	2.27%
			Miyazaki	0.52	25.0%
	[35]	Pred(0.25)	Albrecht	0.46	56.5%
			Miyazaki	0.59	47.5%
			Desharnais	0.51	49.0%
			Miyazaki	0.40	54.0%

## 7. Discussion

This section presents the discussion on the limitations identified from literature, solutions to problem formulations (mentioned in Section 2.4: Problem formulation and research questions) and contributions achieved by this work. Problem formulation identified in this work are listed below:

**I. *Problem***: Absence of accuracy and diversity considerations while creating ensemble.

***Solution*:** The ensemble accuracy and diversity measures are both handled in this work with the incorporation of multi-dimensional bagging (for diversity) and FFA metaheuristics (for accuracy).

For ensuring both diversity and accuracy, divers sets of hyperparameters (coming from divers Sub_SPs) are obtained to train individual bags from the dataset. Besides, best hyperparameters from each Sub_SP are attained. Accuracy is certainly enhanced with the use of FFA in all datasets. Application of FFA in both perspectives (i.e., hyperparameter optimization and ensemble weight optimization) enhanced overall effort prediction accuracy (and Figs 16–23). Mdb schemes established with FFA optimized hyperparameters perform well compared to base algorithms and normal bagging. FFA applied in assigning weights again produces less error measures and increased accuracy. Hence, accuracy measure for proposed model is established.

Considering the diversity measures, it is important for good-performing ensemble to generate different error on any given sample, i.e., models part of ensemble, should produce different error for same sample data [86]. In our proposed Mdb schemes, errors produced by each bag are different due to different hyperparameter configuration each time, hence ensuring diversity.

Ideally, diversity can be sensed when predictions made by each ensemble member are uncorrelated and independent [86]. In our proposed Mdb model, set of hyperparameters are different from one bag to another, i.e., hyperparameter to train one bag can never be the same as other bag since hyperparameters of all bags are coming from different Sub_SPs. This reflects, bags produce while establishing Mdb schemes are highly uncorrelated, ensuing Tier-1 diversity.

Moreover, models in an ensemble may be more dependent if they share the same algorithm, hence halting the diversity. The tier-2 of proposed scheme incorporates MoWE, in which Mdb schemes of three different algorithms (RF, SVR and DeepNet) are combined. All these algorithms came from very diverse implementation style, i.e., RF is bagged version of DTs, SVR is distance-based model while DeepNet entails back propagation learning. Hence, model independence is established, which in turn contributes in ensuring diversity.

Another conclusion about enhanced ensemble performance is that; it is desirable for individual learners constituting ensemble, to be diverse in accuracy as well [86], i.e., ensemble should be an apt mixture of highly accurate learners as well as some low performing learners. The reason is, combining a set of top-performing models will likely deteriorate the result of ensemble since estimation achieved by those models will be highly correlated. Hence combing accurate models with some weak learners make correlation less pertinent [86]. In proposed Mdb scheme, grids of Sub_SPs (Figs 5, 7, 9 and 11), showing some areas of higher error (area shown dark color). This means, hyperparameter coming from these Sub_SPs may generate bags having relatively higher errors. Combining these bags with the ones having less error will create diverse bagging scheme, ensuring minimum correlation among individual learners (i.e., bags).

**II. *Problem*:** Analyzing the impact of both optimization domains (hyperparameter optimization and optimal weights assignment) while creating ensemble is missing.

***Solution*:** This work effectively incorporated optimization in both domains. Hyperparameters of base algorithms are optimized (using FFA) to make Mdb schemes, which are then combined in heterogenous ensemble with optimal weights (optimized with FFA). Here, we can address the RQ1.

### *RQ1*: Does optimization included in both domains (hyperparameter optimization and optimal weights assignment) improve estimation performance?

***Answer*:** As

Answer: As discussed earlier, FFA among all optimization algorithms gives better MAE on all datasets (Appendix A: Table 12 in S1 File). However, even performance of models in Appendix A: Table 12 in S1 File, is not higher than Mdb and MoWE (i.e., models involving FFA-optimized hyperparameters and weights simultaneously) represented in Appendix B in S1 File. As we can see, MAE of all models in Appendix B in S1 File (where both hyperparameter and weight optimization is involved simultaneously) is lower than MAE of all models in Appendix A: Table 12 in S1 File (where only hyperparameter optimization is involved). This reflects, optimization enabled in both aspects i.e., hyperparameters as well as ensemble weights contribute to better performance.

For further highlighting the importance of optimization in both domains, results of Mdb schemes and MoWE can also be compared. As it is evident from the results (Table 8 and Figs 16–23), proposed MoWE technique (including both optimization aspects simultaneously) performed better, compared to Mdb schemes (including hyperparameter optimization only).

It is important to note, all ensembles created without hyperparameter optimization (i.e., Bagging, Stacking, Majority voting, Weighted ensemble) failed to achieve good performance. This also reflects importance of hyperparameter optimization before constructing ensemble. To conclude, models enabled with optimization in both domains achieved better effort prediction in all contexts.

**III. *Problem*:** For ML hyperparameter optimization, no consideration is made on defining search-space selection criteria.

***Solution*:** A well-defined, error-based search space division mechanism is proposed in the study. Creating Sub_SPs from large initial SP and finding best optimizing solution from each Sub_SP helped in improving performance in two regards:

- Facilitates in getting multiple diverse models, eventually enabling diversity in ensemble.
- Providing the chance to include models with slightly higher level of error, so that ensemble members remain uncorrelated and do not contrast the performance of each other (as discussed above).

From this perspective, answer to the RQ2 is given as follows:

### *RQ2*: Does performing search space division endorse same results as utilizing entire search space?

***Answer*:** Utilizing entire search space for creating estimation model is not giving results as good as the models aided with search space division. Appendix A: Table 13 in S1 File listed MAE performance of solo learners, with hyperparameter optimization on entire search space. As it is clear from the table, models are showing visibly larger MAE than all Mdb schemes (Appendix B in S1 File), in which search space division is applied.

**IV. *Problem*:** Investigating the use of both ensemble mechanisms (Homogeneous and Heterogenous) simultaneously is overlooked.

***Solution*:** This study effectively established the effort estimation framework by integrating both types of ensembles. Mdb schemes (homogeneous ensembles), surpassed their base learner as well as normal bagging algorithm. All these accurate Mdb schemes led to further improved MoWE ensemble (Table 8). Wilcoxon statistical test performed on models signifies the same conclusion (Tables 9 and 10).

From this conclusion, RQ3 is address as follows:

### *RQ3*: Does integration of Homogeneous and Heterogenous ensemble tend to improve the performance or Homogeneous/Heterogenous ensemble alone can give good performance?

***Answer*:** Homogeneous ensemble (Mdbs) alone can give good performance, only when compared to their base learners and normal bagging. When Mdb schemes are combined in form of heterogenous ensemble (MoWE), prediction error reduces further. For MoWE, evaluation metrices gave improved results compared to all Mdb schemes, in all datasets (Appendix B in S1 File).

It is important to mention, on some datasets, performance attained by Mdb schemes is not significantly different (at α = 0.05) than their MoWE ensemble, (Section 6.2: Result of statistical evaluation). However, at significance level α = 0.1, MoWE models of all datasets performed significantly higher than their Mdb schemes. Hence, we can conclude, homogeneous ensembles integrated with heterogenous ensemble tend to improve performance of estimation, while in some cases, difference in performance of homogeneous and heterogenous ensemble is not significant on higher α.

## 8. Threats to validity

This section describes threats to validity on the conclusion derived from this work.

*Construct validity*: it is verified by ensuring the reliability of performance measures used in the study. To avoid unfitting measures of the evaluation quality, this study utilizes 5 evaluation metrices (MAE, RMSE, MMRE, MdMRE and PRED) along with SA and effect size, best suited for regression problems and made good compliance with the concept of the study. MMRE criteria, however, is vulnerable for generating biased results as reported by few researches [25]. This is effectively handled by adding unbiased measures of SA and effect size, which are less prone to bias and asymmetry assumption.

*Internal validity*: For this study, RF, SVR, DeepNet are the solo learner choices for ensemble construction. The utilization of said techniques are supported by multiple EEE (Section 2.1: Ensemble effort estimation), and these algorithms provide considerable improvement while applying proposed scheme. However, there is a margin of testing other solo learners to verify if ensemble performance tends to improve. Hence, systematic analysis of other state-of-the-art solo learners is planned to be part of future extension of this work. Another potential threat to ML implementation is the choice of hyperparameters applied to each model. This aspect is effectively handles in this work since a rigorous analysis is made to extract best possible hyperparameter from each model (via search space division and FFA algorithm). For ensemble creation, optimized weights are assigned to each model, according to error improvement it has shown. Hence, threat to assign unjustified weights or unreasonable combination method in ensemble model is avoided.

*External Validity*: This concern is included to verify the validity perimeter of this study’s results. The proposed ensemble scheme works effectively to all ML based effort estimation problems, having definitive set of hyperparameters. Besides this, proposed scheme is evaluated on eight well known datasets of SEACRAFT repository, diverse in terms of software projects and number of features. Further, these datasets contain both numerical and categorical/ordinal features and proposed scheme worked well for deriving prediction from both types of data. Hence this work encompasses sufficiently larger perimeter of estimation.

*Conclusion validity*: Besides the evaluation metrices, the conclusion of the study is also verified by non-parametric Wilcoxon test. We performed Wilcoxon test comparison between *Multi-dimension bagging schemes* over base algorithms and normal bagging to verify the effectiveness of hyperparameter optimization process in generating better versions of base models. Further, statical results are evaluated on significance level of 0.05 to avoid type 1 error and generating any false positives in the evaluation results. Also, 10-fold CV is applied to achieve adequate number of iterations for performing each experimentation. This procedure is sufficient to avoid biasing the results and minimizes sample dependence.

## 9. Conclusion and future work

Software development effort estimation (SDEE) is crucial software project management activity. For this, machine learning (ML) approaches are considered reliable due to the absence of human biasness. However, single ML techniques are sometimes providing variable performance, which can be stabilized using ensemble effort estimation (EEE) methods. Hyperparameter tuning in SDEE plays a vital role in producing more precise results for a ML and for achieving a higher prediction accuracy in ensembles. The reason is, accurate ensembles may require accurate single techniques for better effort prediction. Similarly accurate weights assigned while combining solo learners is important in determining ensemble performance.

This study aims to incorporate both types of ensemble techniques (i.e., Homogeneous and Heterogenous ensemble) for maximizing the performance of estimation. In addition to that, metaheuristic optimization (MO) applied in finding optimal hyperparameter of solo base learners and optimal weights when combining prediction of base algorithms. This work proposed a 2-tier framework for software effort estimation, namely Metaheuristic*-optimized Multi-dimensional bagging scheme and weighted ensemble (MoMdbWE)*. For Tier-1, proposed technique named as *Multi-dimensional bagging scheme (Mdb)* is applied, in which, hyperparameter search space is divided into Sub-Search Spaces (Sub-SPs). This search-space division ensures that best hyperparameters from multiple section of a large search-space are extracted. *Firefly optimization algorithm* is used to get multiple sets of optimized hyperparameters from each Sub_SP. These optimized hyperparameters are used to train bags of base algorithms. Best hyperparameters from each Sub_SP are attained and hyperparameter sets are different for each Sub_SPs, which ensures both diversity and accuracy. Tier-2 of proposed framework is implemented by creating ensemble of three individual Mdb schemes. Optimized weights of each Mdb scheme are obtained with of Firefly algorithm and combined in form of ensemble.

Three most widely used ML techniques (RF, SVR and DeepNet) are implemented as base learners to construct proposed framework. To verify the performance of FFA, best hyperparameters of each base algorithm are obtained from FFA and other optimization techniques (GS, GA, PSO) which confirmed that FFA is most suitable optimization choice. Results of proposed MoMdbWE framework are compared with solo base algorithms and state-of-the-art ensemble techniques; bagging, gradient boosting, stacking, majority voting and weighted ensemble (with non-optimized wights). Performance metrices (MAE, RMSE, MMRE, MdMRE, Pred(0.25), SA and Δ) and statistical evaluation (Wilcoxon test) clearly stated that proposed MoMdbWE has shown considerable improvement in results. Also, a comparative evaluation with previous EEE studies also shows that proposed *MoMdbWE* established more accurate model.

As future work, we intended to replicate this work with other bio-inspired algorithms (Bat algorithm, Artificial bee colony, Ant colony) to check whether more optimized solutions can be achieved. Moreover, we will discuss the implication of proposed framework on data obtained from open source and crowdsourced software environment to ensure the scalability of our proposed model.

## Supporting information

S1 File (DOCX)
