# Supplementary material for: Ensemble effort estimation with metaheuristic hyperparameters and weight optimization for achieving accuracy
Source: PLoS One. 2024 Apr 4;19(4):e0300296. doi: 10.1371/journal.pone.0300296 (PMC10994292; doi:10.1371/journal.pone.0300296)
Supplement: S1 File — (DOCX) [file pone.0300296.s001.docx]

**Appendix A**

**Descriptive statistics of hyperparameter for three base algorithms and performance comparison of MO algorithms**

Table 12: Descriptive statistics of Hyperparameters for Mdb Schemes

|  |  | Min | Max | Mean | Median |
| --- | --- | --- | --- | --- | --- |
| RF | $mtry$ | 2 | 15 | 6 | 5 |
|  | $ntree$ | 105 | 470 | 360 | 257 |
|  | $nodesize$ | 10 | 48 | 34 | 21 |
| SVR | $coef0$ | 0.0042 | 3.9663 | 1.1512 | 0.5644 |
|  | $epsilon$ | 0.1 | 0.9 | 0.566 | 0.6 |
|  | $cost$ | 1 | 100 | 41 | 33 |
|  | $gamma$ | 0.001 | 0.805 | 0.0643 | 0.018 |
| DeepNet | $size$ | 2 | 10 | 6 | 5 |
|  | $decay$ | 0.001 | 0.2 | 0.154 | 0.01 |

Table 13: Performance Comparison of Optimization Algorithms on effort estimation dataset

| Dataset | MO | RF | | SVR | | DeepNet | |
| --- | --- | --- | --- | --- | --- | --- | --- |
|  |  | Fitness(MAE) | Elapsed Time  (sec) | Fitness (MAE) | Elapsed Time (sec) | Fitness (MAE) | Elapsed Time (sec) |
| Albrecht | GS | 0.349 | 116 | 0.356 | 173 | 0.450 | 27 |
|  | GA | 0.265 | 33 | 0.246 | 15 | 0.220 | 18 |
|  | PSO | 0.258 | 12 | 0.222 | 10 | 0.306 | 17 |
|  | FFA | 0.125 | 18 | 0.186 | 16 | 0.209 | 15 |
| Desharnais | GS | 0.147 | 140 | 0.146 | 156 | 0.189 | 59 |
|  | GA | 0.138 | 12 | 0.175 | 17 | 0.169 | 29 |
|  | PSO | 0.193 | 30 | 0.185 | 10 | 0.240 | 35 |
|  | FFA | 0.112 | 11 | 0.143 | 20 | 0.134 | 39 |
| Miyazaki | GS | 0.122 | 152 | 0.210 | 137 | 0.206 | 32 |
|  | GA | 0.027 | 10 | 0.142 | 16 | 0.144 | 23 |
|  | PSO | 0.078 | 25 | 0.172 | 17 | 0.100 | 17 |
|  | FFA | 0.018 | 20 | 0.066 | 16 | 0.084 | 21 |
| China | GS | 0.194 | 590 | 0.315 | 237 | 0.089 | 80 |
|  | GA | 0.018 | 105 | 0.079 | 95 | 0.082 | 45 |
|  | PSO | 0.075 | 89 | 0.113 | 49 | 0.075 | 16 |
|  | FFA | 0.016 | 230 | 0.077 | 349 | 0.071 | 21 |
| Cocomo81 | GS | 0.187 | 207 | 0.430 | 107 | 0.056 | 32 |
|  | GA | 0.017 | 22 | 0.185 | 16 | 0.138 | 13 |
|  | PSO | 0.144 | 25 | 0.204 | 14 | 0.091 | 18 |
|  | FFA | 0.016 | 38 | 0.130 | 19 | 0.019 | 24 |
| Finnish | GS | 0.337 | 56 | 0.510 | 32 | 0.780 | 16 |
|  | GA | 0.223 | 10 | 0.329 | 16 | 0.306 | 13 |
|  | PSO | 0.338 | 25 | 0.275 | 17 | 0.533 | 17 |
|  | FFA | 0.054 | 23 | 0.226 | 16 | 0.304 | 21 |
| Kitchenham | GS | 0.056 | 102 | 0.467 | 38 | 0.056 | 22 |
|  | GA | 0.039 | 11 | 0.034 | 19 | 0.104 | 12 |
|  | PSO | 0.036 | 36 | 0.038 | 17 | 0.071 | 18 |
|  | FFA | 0.028 | 15 | 0.017 | 12 | 0.036 | 28 |
| Maxwell | GS | 0.178 | 147 | 0.410 | 147 | 0.567 | 32 |
|  | GA | 0.116 | 37 | 0.133 | 27 | 0.135 | 13 |
|  | PSO | 0.161 | 48 | 0.139 | 15 | 0.186 | 17 |
|  | FFA | 0.079 | 20 | 0.090 | 19 | 0.127 | 21 |

**Appendix B**

**Performance of base algorithms, Mdb and MoWE using five accuracy measures: MAE, RMSE, MMRE, MdMRE, Pred(0.25)**

Table 14:Results of Proposed Mdb and MoWE on Albrecht dataset

|  | MAE | RMSE | MMRE | MdMRE | Pred |
| --- | --- | --- | --- | --- | --- |
| RF | 0.121 | 0.188 | 0.363 | 0.367 | 0.62 |
| RF_Bagging | 0.121 | 0.194 | 0.39 | 0.407 | 0.62 |
| RF_Mdb | 0.118 | 0.185 | 0.325 | 0.31 | 0.74 |
| SVR | 0.181 | 0.199 | 0.45 | 0.45 | 0.68 |
| SVR_Bagging | 0.184 | 0.311 | 0.498 | 0.501 | 0.7 |
| SVR_Mdb | 0.168 | 0.282 | 0.428 | 0.408 | 0.87 |
| DeepNet | 0.08 | 0.125 | 0.335 | 0.344 | 0.64 |
| DeepNet_Bagging | 0.112 | 0.23 | 0.359 | 0.333 | 0.52 |
| DeepNet_Mdb | 0.076 | 0.099 | 0.276 | 0.283 | 0.68 |
| Gradient Boosting | 0.131 | 0.199 | 0.337 | 0.373 | 0.68 |
| Stacking | 0.137 | 0.311 | 0.361 | 0.41 | 0.67 |
| Majority Voting | 0.118 | 0.282 | 0.279 | 0.31 | 0.69 |
| Weighted ensemble | 0.134 | 0.478 | 0.423 | 0.231 | 0.64 |
| Proposed MoWE | 0.067 | 0.096 | 0.232 | 0.172 | 0.88 |

Table 15: Results of Proposed Mdb and MoWE on Desharnais dataset

|  | MAE | RMSE | MMRE | MdMRE | Pred |
| --- | --- | --- | --- | --- | --- |
| RF | 0.108 | 0.164 | 0.84 | 0.403 | 0.67 |
| RF_Bagging | 0.108 | 0.167 | 0.919 | 0.436 | 0.62 |
| RF_Mdb | 0.099 | 0.141 | 0.847 | 0.396 | 0.71 |
| SVR | 0.136 | 0.224 | 0.834 | 0.413 | 0.71 |
| SVR_Bagging | 0.117 | 0.201 | 1.114 | 0.398 | 0.76 |
| SVR_Mdb | 0.117 | 0.199 | 0.948 | 0.375 | 0.77 |
| DeepNet | 0.165 | 0.256 | 1.75 | 0.303 | 0.62 |
| DeepNet_Bagging | 0.138 | 0.204 | 0.939 | 0.414 | 0.65 |
| DeepNet_Mdb | 0.126 | 0.181 | 0.671 | 0.354 | 0.67 |
| Gradient Boosting | 0.165 | 0.164 | 0.834 | 0.413 | 0.68 |
| Stacking | 0.138 | 0.167 | 1.114 | 0.298 | 0.61 |
| Majority Voting | 0.126 | 0.141 | 0.948 | 0.375 | 0.64 |
| Weighted ensemble | 0.145 | 0.156 | 1.158 | 0.345 | 0.6 |
| Proposed MoWE | 0.066 | 0.096 | 0.306 | 0.296 | 0.95 |

Table 16: Results of Proposed Mdb and MoWE on Miyazaki dataset

|  | MAE | RMSE | MMRE | MdMRE | Pred |
| --- | --- | --- | --- | --- | --- |
| RF | 0.015 | 0.026 | 5.26 | 0.732 | 0.73 |
| RF_Bagging | 0.015 | 0.02 | 1.245 | 0.643 | 0.67 |
| RF_ Mdb | 0.011 | 0.017 | 1.241 | 0.413 | 0.8 |
| SVR | 0.013 | 0.023 | 4.98 | 0.533 | 0.6 |
| SVR_Bagging | 0.014 | 0.032 | 1.631 | 0.515 | 0.67 |
| SVR_ Mdb | 0.013 | 0.018 | 1.794 | 0.341 | 0.8 |
| DeepNet | 0.02 | 0.04 | 1.286 | 0.523 | 0.4 |
| DeepNet_Bagging | 0.044 | 0.138 | 5.818 | 0.876 | 0.74 |
| DeepNet_ Mdb | 0.017 | 0.037 | 1.186 | 0.425 | 0.76 |
| Gradient Boosting | 0.013 | 0.023 | 1.186 | 0.732 | 0.63 |
| Stacking | 0.014 | 0.018 | 1.286 | 0.643 | 0.6 |
| Majority Voting | 0.011 | 0.018 | 5.818 | 0.413 | 0.57 |
| Weighted ensemble | 0.103 | 0.032 | 0.905 | 0.516 | 0.68 |
| Proposed MoWE | 0.008 | 0.014 | 0.43 | 0.39 | 0.87 |

Table 17: Results of Proposed Mdb and MoWE on China dataset

|  | MAE | RMSE | MMRE | MdMRE | Pred |
| --- | --- | --- | --- | --- | --- |
| RF | 0.017 | 0.067 | 0.225 | 0.148 | 0.73 |
| RF_Bagging | 0.019 | 0.069 | 0.261 | 0.175 | 0.65 |
| RF_ Mdb | 0.013 | 0.061 | 0.149 | 0.059 | 0.68 |
| SVR | 0.025 | 0.089 | 0.286 | 0.147 | 0.67 |
| SVR_Bagging | 0.025 | 0.088 | 0.300 | 0.163 | 0.61 |
| SVR_ Mdb | 0.014 | 0.026 | 0.569 | 0.197 | 0.55 |
| DeepNet | 0.030 | 0.064 | 0.871 | 0.422 | 0.31 |
| DeepNet_Bagging | 0.037 | 0.099 | 0.832 | 0.433 | 0.25 |
| DeepNet_ Mdb | 0.012 | 0.025 | 0.256 | 0.135 | 0.65 |
| Gradient Boosting | 0.022 | 0.072 | 0.456 | 0.202 | 0.56 |
| Stacking | 0.016 | 0.061 | 0.332 | 0.179 | 0.61 |
| Majority Voting | 0.042 | 0.096 | 0.474 | 0.490 | 0.10 |
| Weighted ensemble | 0.064 | 0.131 | 0.822 | 0.830 | 0.10 |
| Proposed MoWE | 0.011 | 0.021 | 0.362 | 0.187 | 0.80 |

Table 18: Results of Proposed Mdb and MoWE on Cocomo81 dataset

|  | MAE | RMSE | MMRE | MdMRE | Pred |
| --- | --- | --- | --- | --- | --- |
| RF | 0.032 | 0.041 | 84.844 | 7.327 | 0.24 |
| RF_Bagging | 0.038 | 0.052 | 69.174 | 8.140 | 0.10 |
| RF_Mdb | 0.011 | 0.017 | 22.515 | 2.548 | 0.32 |
| SVR | 0.025 | 0.037 | 37.749 | 4.531 | 0.21 |
| SVR_Bagging | 0.029 | 0.042 | 90.917 | 6.610 | 0.21 |
| SVR_Mdb | 0.009 | 0.009 | 11.291 | 0.668 | 0.42 |
| DeepNet | 0.025 | 0.043 | 43.197 | 0.863 | 0.21 |
| DeepNet_Bagging | 0.016 | 0.020 | 21.639 | 1.369 | 0.16 |
| DeepNet_Mdb | 0.014 | 0.016 | 2.921 | 0.462 | 0.21 |
| Gradient Boosting | 0.045 | 0.052 | 583.036 | 4.663 | 0.11 |
| Stacking | 0.035 | 0.044 | 182.224 | 3.217 | 0.15 |
| Majority Voting | 0.013 | 0.020 | 10.002 | 0.622 | 0.15 |
| Weighted ensemble | 0.016 | 0.026 | 3.643 | 0.867 | 0.20 |
| Proposed MoWE | 0.006 | 0.021 | 16.788 | 4.180 | 0.78 |

Table 19: Results of Proposed Mdb and MoWE on Finnish dataset

|  | MAE | RMSE | MMRE | MdMRE | Pred |
| --- | --- | --- | --- | --- | --- |
| RF | 0.097 | 0.124 | 0.853 | 0.132 | 0.67 |
| RF_Bagging | 0.097 | 0.120 | 0.802 | 0.157 | 0.67 |
| RF_Mdb | 0.039 | 0.040 | 0.230 | 0.034 | 0.75 |
| SVR | 0.110 | 0.147 | 0.911 | 0.187 | 0.58 |
| SVR_Bagging | 0.120 | 0.160 | 1.021 | 0.175 | 0.58 |
| SVR_Mdb | 0.038 | 0.063 | 0.437 | 0.030 | 0.83 |
| DeepNet | 0.059 | 0.074 | 0.461 | 0.097 | 0.75 |
| DeepNet_Bagging | 0.064 | 0.077 | 0.454 | 0.120 | 0.75 |
| DeepNet_Mdb | 0.046 | 0.065 | 0.456 | 0.057 | 0.75 |
| Gradient Boosting | 0.429 | 0.541 | 0.937 | 0.943 | 0.10 |
| Stacking | 0.058 | 0.068 | 0.477 | 0.095 | 0.67 |
| Majority Voting | 0.234 | 0.287 | 0.505 | 0.504 | 0.30 |
| Weighted ensemble | 0.385 | 0.473 | 0.767 | 0.830 | 0.20 |
| Proposed MoWE | 0.027 | 0.057 | 0.402 | 0.055 | 0.88 |

Table 20: Results of Proposed Mdb and MoWE on Kitchenham dataset

|  | MAE | RMSE | MMRE | MdMRE | Pred |
| --- | --- | --- | --- | --- | --- |
| RF | 0.024 | 0.136 | 0.536 | 0.216 | 0.52 |
| RF_Bagging | 0.024 | 0.135 | 0.528 | 0.231 | 0.52 |
| RF_Mdb | 0.022 | 0.133 | 0.197 | 0.083 | 0.75 |
| SVR | 0.026 | 0.146 | 0.640 | 0.253 | 0.48 |
| SVR_Bagging | 0.026 | 0.147 | 0.567 | 0.252 | 0.50 |
| SVR_Mdb | 0.005 | 0.031 | 0.089 | 0.016 | 0.93 |
| DeepNet | 0.024 | 0.134 | 0.640 | 0.221 | 0.55 |
| DeepNet_Bagging | 0.023 | 0.129 | 0.525 | 0.202 | 0.55 |
| DeepNet_Mdb | 0.030 | 0.130 | 2.602 | 1.126 | 0.20 |
| Gradient Boosting | 0.025 | 0.140 | 0.463 | 0.209 | 0.55 |
| Stacking | 0.019 | 0.102 | 0.391 | 0.211 | 0.52 |
| Majority Voting | 0.029 | 0.143 | 0.525 | 0.493 | 0.22 |
| Weighted ensemble | 0.034 | 0.149 | 0.829 | 0.831 | 0.10 |
| Proposed MoWE | 0.004 | 0.006 | 0.090 | 0.019 | 0.95 |

Table 21: Results of Proposed Mdb and MoWE on Maxwell dataset

|  | MAE | RMSE | MMRE | MdMRE | Pred |
| --- | --- | --- | --- | --- | --- |
| RF | 0.093 | 0.191 | 0.586 | 0.290 | 0.42 |
| RF_Bagging | 0.101 | 0.200 | 0.684 | 0.382 | 0.37 |
| RF_Mdb | 0.086 | 0.188 | 0.506 | 0.230 | 0.53 |
| SVR | 0.108 | 0.225 | 0.520 | 0.347 | 0.37 |
| SVR_Bagging | 0.109 | 0.225 | 0.640 | 0.356 | 0.37 |
| SVR_Mdb | 0.043 | 0.112 | 0.130 | 0.104 | 0.84 |
| DeepNet | 0.095 | 0.166 | 0.690 | 0.427 | 0.16 |
| DeepNet_Bagging | 0.117 | 0.231 | 0.768 | 0.484 | 0.21 |
| DeepNet_Mdb | 0.056 | 0.126 | 0.401 | 0.115 | 0.74 |
| Gradient Boosting | 0.100 | 0.217 | 0.704 | 0.308 | 0.42 |
| Stacking | 0.094 | 0.187 | 0.384 | 0.335 | 0.32 |
| Majority Voting | 0.125 | 0.216 | 0.555 | 0.570 | 0.15 |
| Weighted ensemble | 0.166 | 0.272 | 0.840 | 0.857 | 0.30 |
| Proposed MoWE | 0.040 | 0.125 | 0.082 | 0.036 | 0.89 |
